# Antibacterial Ingredients and Modes of the Methanol-Phase Extract from the Fruit of *Amomum villosum* Lour.

**DOI:** 10.3390/plants13060834

**Published:** 2024-03-14

**Authors:** Kaiyue Zhang, Fengfeng Cao, Yueliang Zhao, Hengbin Wang, Lanming Chen

**Affiliations:** 1 Key Laboratory of Quality and Safety Risk Assessment for Aquatic Products on Storage and Preservation, Ministry of Agriculture and Rural Affairs of the People’s Republic of China, Shanghai 201306, China; 2 College of Food Science and Technology, Shanghai Ocean University, Shanghai 201306, China; 3 Department of Internal Medicine, Division of Hematology, Oncology, and Palliative Care, Massey Cancer Center, School of Medicine, Virginia Commonwealth University, Richmond, VA 23298, USA

**Keywords:** *Amomum villosum* Lour., antibacterial compound, antibacterial mechanism, pathogen, infectious disease, pharmacophagous plant

## Abstract

Epidemics of infectious diseases threaten human health and society stability. Pharmacophagous plants are rich in bioactive compounds that constitute a safe drug library for antimicrobial agents. In this study, we have deciphered for the first time antibacterial ingredients and modes of the methanol-phase extract (MPE) from the fruit of *Amomum villosum* Lour. The results have revealed that the antibacterial rate of the MPE was 63.64%, targeting 22 species of common pathogenic bacteria. The MPE was further purified by high performance liquid chromatography (Prep-HPLC), and three different constituents (Fractions 1–3) were obtained. Of these, the Fraction 2 treatment significantly increased the cell membrane fluidity and permeability, reduced the cell surface hydrophobicity, and damaged the integrity of the cell structure, leading to the leakage of cellular macromolecules of Gram-positive and Gram-negative pathogens (*p* < 0.05). Eighty-nine compounds in Fraction 2 were identified by ultra HPLC-mass spectrometry (UHPLC-MS) analysis, among which 4-hydroxyphenylacetylglutamic acid accounted for the highest 30.89%, followed by lubiprostone (11.86%), miltirone (10.68%), and oleic acid (10.58%). Comparative transcriptomics analysis revealed significantly altered metabolic pathways in the representative pathogens treated by Fraction 2 (*p* < 0.05), indicating multiple antibacterial modes. Overall, this study first demonstrates the antibacterial activity of the MPE from the fruit of *A. villosum* Lour., and should be useful for its application in the medicinal and food preservative industries against common pathogens.

## 1. Introduction

Epidemics of infectious diseases threaten human health, cause loss of life, and seriously impact the economy [1,2]. In recent decades, due to the inappropriate use of antibiotics, clinical antibiotic therapy has become increasingly ineffective in preventing outbreaks and spreading of infectious diseases [3]. Therefore, it is imperative to search for safe and effective antimicrobial alternatives. Pharmacophagous plants with safety and low toxicity properties are traditionally used to treat many diseases. These plant extracts constitute an ideal drug library for antimicrobial agents [4].

*Amomum villosum* Lour. is a comestible medicinal plant that belongs to the Zingiberaceae family. This plant is mainly distributed in the tropical regions of Asia and Oceania. Its dry fruits and seeds are often used as cooking condiments, with a unique and rich aroma, and also used as valuable traditional medicines, such as for the obstruction of body dampness and turbidity, stomach deficiency and cold, and vomiting and diarrhea [5,6]. Recent studies have provided experimental evidence for the pharmacological activities of *A. villosum* Lour., such as anti-ulcer, anti-diarrhea, and anti-inflammation [7]. For example, Yin et al. [8] reported that the extracted labdane and norlabdane diterpenoids from the rhizomes of *A. villosum* Lour. showed anti-inflammatory and α-glucosidase inhibitory activities. Luo et al. [9] reported that the dietary supplement of *A. villosum* Lour. polysaccharide attenuated ulcerative colitis of balb/c mice, a prospective nutritional strategy for the treatment of inflammatory bowel diseases.

Nevertheless, the current literature on the bacteriostasis activity of *A. villosum* Lour. is rare. To the best of our knowledge, the only other study reported that essential oil (EO) of *A. villosum* Lour. inhibited the growth of *Staphylococcus aureus* ATCC43 [10]. In order to further explore the antibacterial activity of *A. villosum* Lour., in this study, we aimed to extract bioactive compounds in the fruit of *A. villosum* Lour. using the methanol–chloroform extraction (M–CE) method, which has been well established in our laboratory [11,12,13,14], and to decipher the antibacterial ingredients and modes of the methanol-phase extract (MPE) from *A. villosum* Lour.

## 2. Results and Discussion

### 2.1. Antibacterial Activity of Crude Extracts from the Fruit of A. villosum Lour.

Based on our recent studies [11,12,13,14], the M–CE method was employed to extract antibacterial substances from the fruit of *A. villosum* Lour. Its water loss rate was 73.68% after freeze-drying at −80 °C for 48 h. The extraction yields of the MPE and chloroform-phase extract (CPE) of *A. villosum* Lour. were 12.00% and 2.80%, respectively.

The antibacterial activities of the MPE and CPE of *A. villosum* Lour. were determined, targeting 22 species of common pathogenic bacteria. As shown in Table 1, the MPE showed an inhibition rate of 63.64% and repressed the growth of 14 species of bacteria, including the following: 2 species of Gram-positive bacteria: *S. aureus* and *Bacillus cereus*; and 12 species of Gram-negative bacteria: *Aeromonas hydrophila*, *Pseudomonas aeruginosa*, *Shigella dysenteriae*, *Shigella flexneri*, *Shigella sonnei*, *Salmonella enterica* subsp. *enterica* (*ex* Kauffmann and Edwards), *Vibrio alginolyticus*, *Vibrio cholerae*, *Vibrio harveyi*, *Vibrio metschnikovi*, *Vibrio mimicus*, and *Vibrio parahaemolyticus*. The CPE showed an inhibition rate of 54.55% and inhibited 1 species of Gram-positive and 11 species of Gram-negative bacteria (Table 1).

Tang et al. [10] used water as a solvent to extract the EO of *A. villosum* Lour. and found its inhibitory effect on *S. aureus* ATCC43. In this study, our results indicated that the M–CE method was more effective in extracting antibacterial substances in *A. villosum* Lour. than water.

Given the higher antibacterial rate (63.64%), the MPE of *A. villosum* Lour. was subjected to further analysis in this study. The minimum inhibitory concentrations (MICs) of the MPE were determined, targeting the 14 species of pathogenic bacteria. As shown in Table 1, the MICs of the MPE ranged from 128 to 1024 μg/mL. The most effective inhibition was observed against the Gram-positive bacterium *S*. *aureus* GIM1.441 and the Gram-negative bacterium *V*. *parahaemolyticus* B2-28, with MICs of 128 μg/mL and 256 μg/mL, respectively (Table 1).

### 2.2. Purification of the MPE of A. villosum Lour.

**Figure 1:**
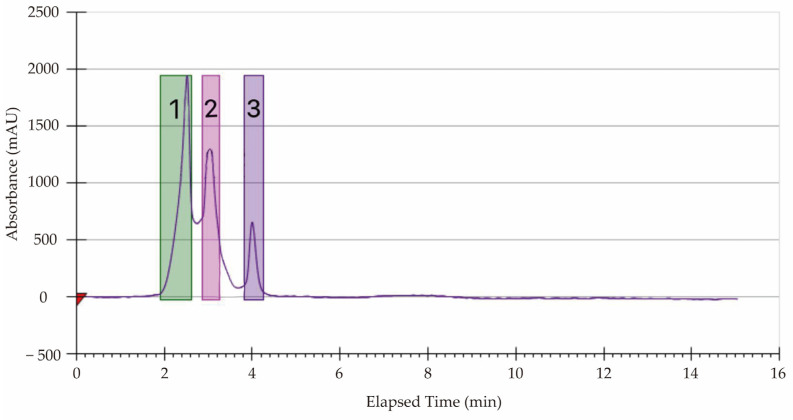
Prep-HPLC diagram of purifying the MPE from the fruit of *A. villosum* Lour. (1–3): the Fraction 1, Fraction 2, and Fraction 3, respectively.

The MPE of *A. villosum* Lour. was prepared in large quantities using the Prep-high-performance-liquid-chromatography (Prep-HPLC) technique. As shown in Figure 1, three distinct constituents (designated as Fractions 1–3) were observed at 1.9–4.2 min when scanning at OD_211_ for 15 min.

**Figure 2:**
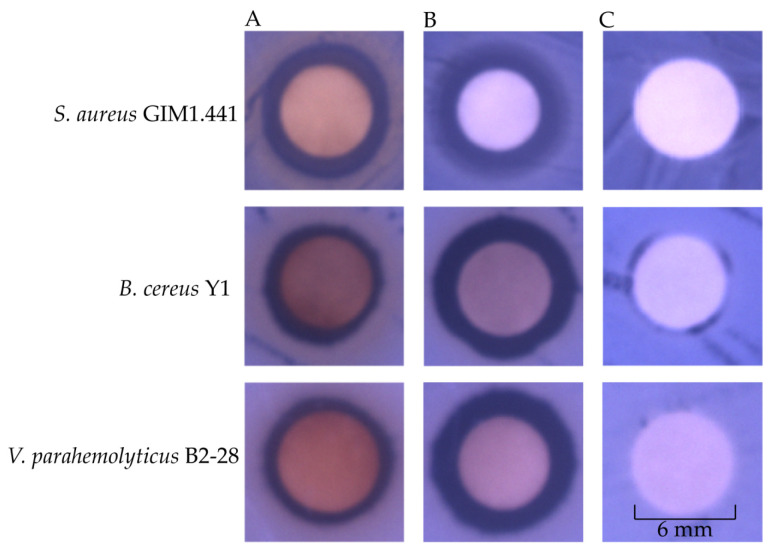
The DIZs of the MPE and Fraction 2 of MPE from *A. villosum* Lour. (**A**–**C**): the MPE, Fraction 2 of MPE, and negative control, respectively.

The antibacterial activity of the three different Fractions was further determined, and the results are presented in Table 2 and Figure 2. Fraction 2 displayed inhibitory effects on the two species of Gram-positive and eight species of Gram-negative bacteria. The diameters of the inhibitory zone (DIZ) ranged between 7 and 11.5 mm. In contrast, Fraction 1 and Fraction 3 had weak and no inhibitory effects, respectively (Table 2).

We also determined the MICs of Fraction 2. As shown in Table 2, the strongest antibacterial efficacy of Fraction 2 was observed against *S. aureus* GIM1.441, *B. cereus* Y1, and *V. parahemolyticus* B2-28, with MICs of 256 μg/mL, 512 μg/mL, and 512 μg/mL, respectively, consistent with the results yielded from the MPE of *A. villosum* Lour.

The Gram-positive bacterium *S. aureus* can cause human skin and tissue infections and sepsis in severe cases [15], while *B. cereus* can cause self-limiting emetic and diarrhea diseases [16]. The Gram-negative bacterium *V. parahemolyticus* is a leading sea-food-borne pathogen worldwide, and common clinical symptoms include headache, nausea, vomiting, and diarrhea. Severe infections caused by *V. parahemolyticus* can develop into sepsis or even death [17].

In order to decipher the antibacterial mechanisms of Fraction 2 of *A. villosum* Lour., based on the above results, the Gram-positive bacteria *S. aureus* GIM1.441 and *B. cereus* Y1 and the Gram-negative bacterium *V. parahemolyticus* B2-28 were chosen as target strains for further analyses in this study.

### 2.3. Inhibited Growth of the Target Strains Treated with Fraction 2 of A. villosum Lour.

We determined growth curves of the three target strains treated with Fraction 2 of *A. villosum* Lour. As shown in Figure S1, *S. aureus* GIM1.441 was strongly inhibited when incubated in tryptone soybean broth (TSB) medium supplemented with 1 x MIC of Fraction 2. The inhibition showed a concentration-dependent mode, as *S. aureus* GIM1.441 was found to grow at 1/2 x MIC of Fraction 2, but showed lower biomass (maximum OD_600_ = 0.9065), as compared to the control group (maximum OD _600_ = 1.205) (Figure S1A).

Similarly, the inhibition by the 1 x MIC of Fraction 2 was also stronger on *B. cereus* Y1 than the 1/2 x MIC (Figure S1B). The same case was apparent for the Gram-negative bacterium *V. parahemolyticus* B2-28, but this bacterium appeared to be the most sensitive to the Fraction 2 treatment among the test strains (Figure S1C).

Taken together, the 1 x MIC (256 μg/mL, 512 μg/mL, 512 μg/mL) of Fraction 2 was chosen as the treatment conditions for *S. aureus* GIM1.441, *B. cereus* Y1, and *V. parahemolyticus* B2-28, respectively, in the further analyses in this study.

### 2.4. Changed Cell Surface Hydrophobicity (CSH), Cell Membrane Fluidity (CMF), and Cell Membrane Permeability (CMP) of the Target Strains Treated with Fraction 2 of A. villosum Lour.

**Figure 3:**
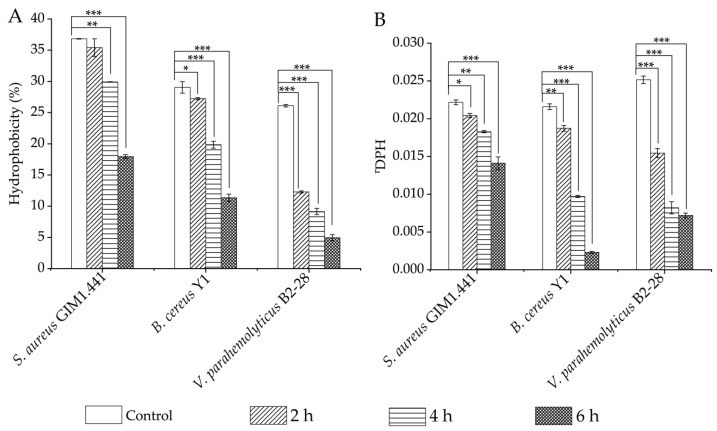
Effects of Fraction 2 (1 x MIC) of *A. villosum* Lour. on the CSH and CMF of the target strains. (**A**,**B**): CSH and CMF, respectively. *: *p* < 0.05; **: *p* < 0.01; ***: *p* < 0.001.

The interaction between microbial cells and the host is influenced by the biophysical properties of the cell membrane, such as the CSH [18]. In this study, we observed that the CSH of the target strains was remarkably reduced in all of the treatment groups after 4 h- and 6 h-treatment of Fraction 2 of *A. villosum Lour.*, as compared to the control groups (*p* < 0.05). Moreover, the decreased CSH of the strains was closely related to prolonged treatment time (Figure 3A). For example, after being treated with Fraction 2 for 2 h, the CSH of *S. aureus* GIM1.441 did not significantly change (*p* > 0.05). However, the decreased CSH was observed to be 1.23-fold and 2.05-fold after treatment for 4 h and 6 h, respectively (*p* < 0.001). Similarly, the CSH of *B. cereus* Y1 decreased by 1.07-fold to 2.56-fold after the treatment for 2 h to 6 h (*p* < 0.05). The strongest decrease (2.12-fold) in the CSH was found in *V*. *parahaemolyticus* B2-28 after being treated with Fraction 2 for 2 h (*p* < 0.001).

The CMF is also a key parameter of the bacterial cell membrane. In this study, 1,6-diphenyl-1,3,5-hexatriene (DPH) was used as a probe to detect the changes in CMF of the target strains. Higher DPH values indicated weaker CMF [19]. As shown in Figure 3B, as compared to the control groups, the CMF of the three target strains increased significantly (1.09-fold, 1.15-fold, and 1.63-fold) after being treated with Fraction 2 for 2 h (*p* < 0.05). Among the three strains, the CMF of *V. parahaemolyticus* B2-28 and *B. cereus* Y1 increased the most (3.06-fold and 9.35-fold) after the 4-h- and 6-h-treatment, respectively (*p* < 0.001).

**Figure 4:**
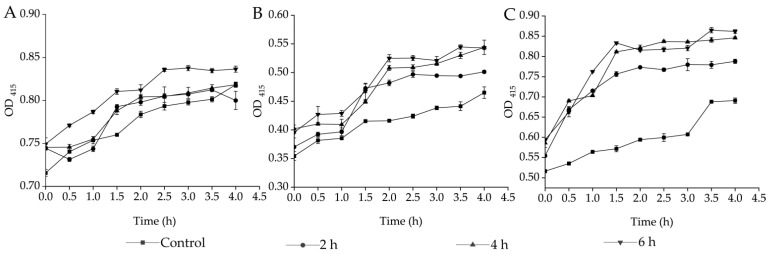
Effects of Fraction 2 of *A. villosum* Lour. on the CMP of the target strains. (**A**–**C**): *S. aureus* GIM1.441, *B. cereus* Y1, and *V. parahemolyticus* B2-28, respectively.

The bacterial cell membrane is a permeable barrier against external harmful substances; therefore, it is the therapeutic target of antimicrobial agents [20]. In this study, the o-nitrophenyl-β-D-galactopyranoside (ONPG) was used as a probe to detect the changes in the CMP of the target strains. As shown in Figure 4A, as compared to the control group, there were no significant changes in the CMP of *S. aureus* GIM1.441 after being treated with Fraction 2 of *A. villosum* Lour. for 2 h and 4 h (*p* > 0.05). However, a significant increase in the CMP was observed after the 6 h-treatment (*p* < 0.05). *B. cereus* Y1 showed a 1.08-fold increase in the CMP after the treatment for 2 h (*p* < 0.05, Figure 4B). Similarly, the increased CMP of *V. parahemolyticus* B2-28 was also observed to be 1.14-fold to 1.25-fold after being treated with Fraction 2 for 2 h to 6 h (*p* < 0.05, Figure 4C).

Taken together, the Fraction 2 treatment can significantly reduce the CSH but increase the CMP and CMF of Gram-positive *S. aureus* GIM1.441 and *B. cereus* Y1 and Gram-negative *V. parahaemolyticus* B2-28. The antibacterial effects are exerted with the treatment-time-dependent mode.

### 2.5. Changed Cell Morphological Structure of the Target Strains Treated with Fraction 2 of A. villosum Lour.

**Figure 5:**
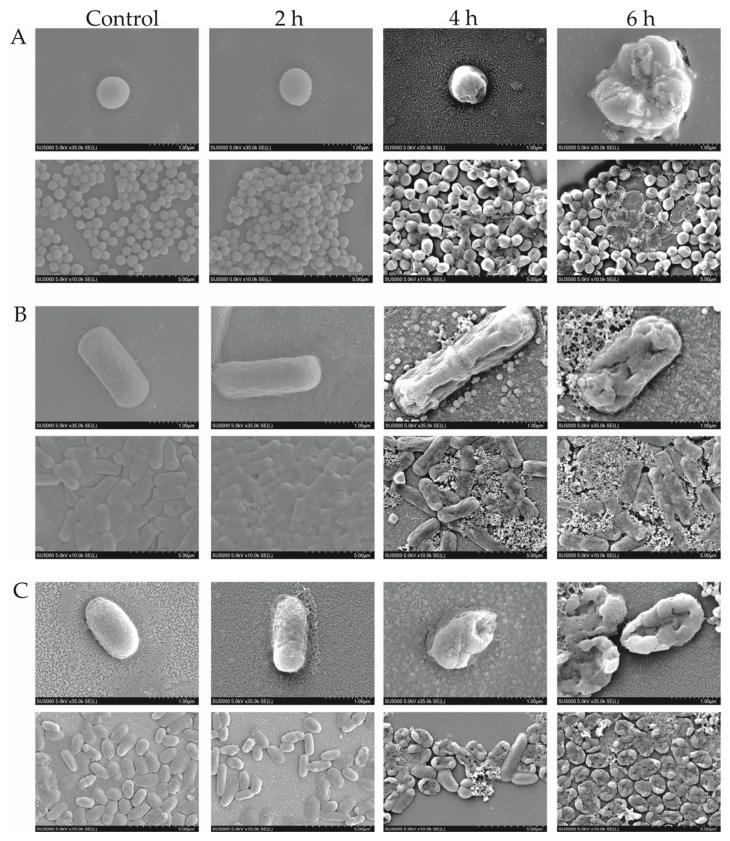
SEM observation of cell structure of the target strains treated with Fraction 2 of *A. villosum* Lour. (**A**–**C**): *S. aureus* GIM1.441, *B. cereus* Y1, and *V. parahemolyticus* B2-28, respectively.

Based on the above results, we wondered whether the cell structure of the target strains was damaged by the Fraction 2 treatment. Therefore, we observed the cell structure changes in *S. aureus* GIM1.441, *B. cereus* Y1, and *V. parahaemolyticus* B2-28 using a scanning electron microscope (SEM). As shown in Figure 5, the bacterial cells in the control groups were intact, with a full shape and clear structure. However, in the treatment groups, the cells showed varying degrees of folds, breaks, and pores after being treated with Fraction 2 for 2 h to 6 h.

For example, for the Gram-positive bacterium *S. aureus* GIM1.441, no significant change in the bacterial cell surface was observed after the 2 h-treatment with Fraction 2. However, the wrinkled cell surface occurred after the 4 h-treatment, and they even burst after the 6 h-treatment (Figure 5A). A similar case was found for *B. cereus* Y1 (Figure 5B).

For the Gram-negative bacterium *V. parahemolyticus* B2-28, severe ruffling on the cell surface, and even a wrinkled cell structure, were observed after the treatment for 2 h. Remarkably, the bacterial cells were fully ruptured after the treatment for 6 h (Figure 5C).

Tang et al. [10] reported that the surface of *S. aureus* ATCC43 appeared irregular, wrinkled, and uneven, but did not burst after being treated with the EO of *A. villosum* Lour. for 6 h. These results have provided additional evidence to validate that the MPE of *A. villosum* Lour. has a stronger antibacterial efficacy on the target strains than the EO.

Taken together, Fraction 2 of *A. villosum* Lour. can destroy the cell structure of Gram-positive and Gram-negative bacteria to varying degrees. Moreover, the treatment is the most effective against the Gram-negative bacterium *V. parahemolyticus* B2-28.

### 2.6. Nucleotide Acid and Protein Exudation of the Target Strains Treated with Fraction 2 of A. villosum Lour.

**Figure 6:**
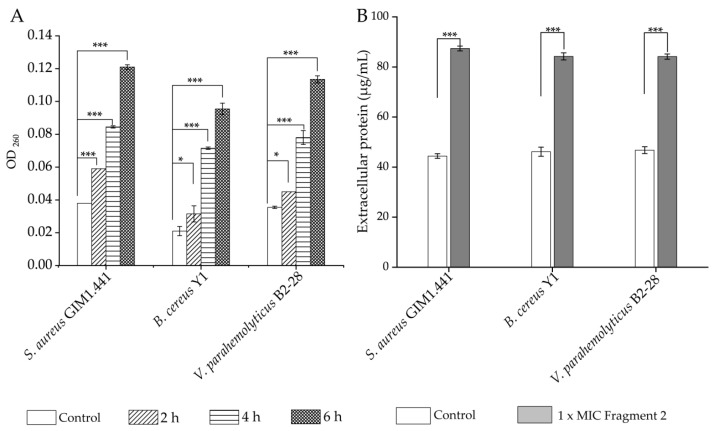
Effects of Fraction 2 of *A. villosum* Lour. on nucleotide acid and protein exudation of the target strains. (**A**,**B**): nucleotide acids and proteins, respectively. *: *p* < 0.05; and ***: *p* < 0.001.

Damage to the cell structure may lead to the leakage of macromolecules. Therefore, we examined the nucleotide acid and protein exudation of the target strains treated with Fraction 2 of *A. villosum Lour*. As shown in Figure 6A, as compared to the control group, the amount of nucleotide acids exuded from *S. aureus* GIM1.441 was significantly increased by 1.55-fold after being treated with Fraction 2 for 2 h (*p* < 0.001). More extracellular nucleotide acids (2.22-fold and 3.18-fold) were detected with the longer treatment time (4 h and 6 h) (*p* < 0.001). A similar case was observed in *B. cereus* Y1 and *V. parahaemolyticus* B2-28.

As shown in Figure 6B, the number of extracellular proteins of *S. aureus* GIM1.441 was also significantly increased by 1.97-fold after being treated with Fraction 2 for 24 h (*p* < 0.001). Likewise, the proteins were exuded by 1.82-fold and 1.80-fold from *B. cereus* Y1 and *V. parahemolyticus* B2-28, respectively, after the Fraction 2 treatment (*p* < 0.001).

Bouyahya et al. [21] reported that the EO of *Origanum compactum* was involved in the changed membrane permeability and leakage of macromolecules. In this study, it can be concluded that the treatment with Fraction 2 of *A. villosum* Lour. results in the leakage of nucleotide acids and proteins from the Gram-positive and Gram-negative strains, consistent with the damaged bacterial cell structure observed with the SEM.

### 2.7. The Altered Metabolic Pathways in the Target Strains Treated with Fraction 2 of A. villosum Lour.

In order to obtain insights into the changes in gene expression at the whole-genome level, we determined the transcriptomes of *S*. *aureus* GIM1.441, *B*. *cereus* Y1, and *V*. *parahaemolyticus* B2-28 treated with Fraction 2 (1 x MIC) for 6 h. The lists of the differential expressed genes (DEGs) in the three strains were deposited in the NCBI SRA database (https://sub-mit.ncbi.nlm.nih.gov/subs/bioproject/, accessed on 25 September 2023) under the accession number PRJNA1020669.

#### 2.7.1. The Altered Metabolic Pathways in *S. aureus* GIM1.441 Treated with Fraction 2 of *A. villosum* Lour.

**Figure 7:**
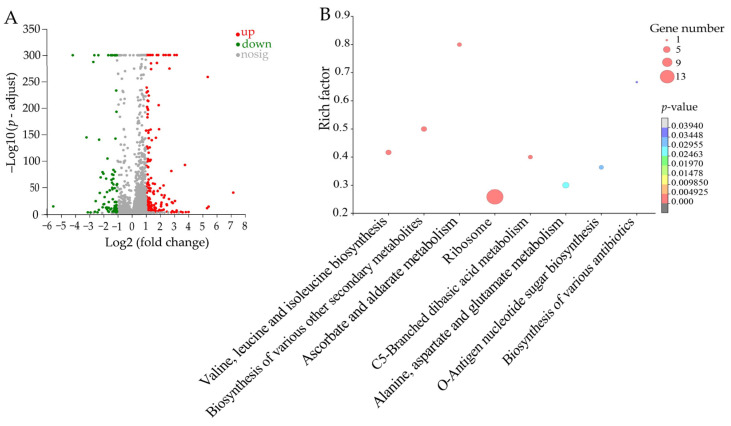
The eight significantly altered metabolic pathways in *S. aureus* GIM1.441 treated with Fraction 2 of *A. villosum* Lour. (**A**,**B**): Volcano plot of the DGEs and the changed metabolic pathways, respectively.

The DEGs in the treatment group accounted for 11.12% (291/2617) of the *S. aureus* GIM1.441 genes, as compared to the control group. Of these, 91 DEGs showed lower transcription levels (fold change (FC) ≤ 0.5), whereas 200 DEGs were up-regulated (FC ≥ 2.0). Eight metabolic pathways were significantly altered in *S. aureus* GIM1.441, including valine, leucine, and isoleucine biosynthesis; the biosynthesis of various other secondary metabolites; ascorbate and aldarate metabolism; C5-branched dibasic acid metabolism; alanine, aspartate, and glutamate metabolism; o-antigen nucleotide sugar biosynthesis; ribosome; and the biosynthesis of various antibiotics (Figure 7, Table S1).

For example, the expression of three DEGs in valine, leucine, and isoleucine biosynthesis was significantly down-regulated (0.39- to 0.447-fold) at the transcriptional levels in *S. aureus* GIM1.441 after being treated with Fraction 2 of *A. villosum* Lour. (*p* < 0.05). Of these, the ketol-acid reductoisomerase (*B4602_RS10775*) was significantly repressed (0.447-fold) (*p* < 0.05), which plays a crucial role in the biosynthesis pathway of branched-chain amino acids [22]. The 2-isopropylmalate synthase (*B4602_RS10780*) was also significantly down-regulated (0.39-fold) (*p* < 0.05), which catalyzes the first step of leucine biosynthesis [23]. The threonine ammonia-lyase IlvA (*B4602_RS10800*) was significantly inhibited as well (0.394-fold) (*p* < 0.05), which has been reported to resist the feedback inhibition of l-isoleucine [24].

In the C5 branched-chain binary acid metabolism, three DEGs were also significantly down-regulated (0.303- to 0.414-fold) (*p* < 0.05). For example, the 3-isopropylmalate dehydrogenase (*B4602_RS10785*) was significantly inhibited (0.379-fold) (*p* < 0.05), which serves as the third specific enzyme for the synthesis of leucine in microorganisms and plants [25].

In alanine, aspartate, and glutamate metabolism, the expression of two DEGs was also significantly down-regulated (0.413- to 0.472-fold) in *S. aureus* GIM1.441 (*p* < 0.05). For example, the adenylosuccinate synthase (*B4602_RS00095*) was significantly inhibited (0.413-fold), which catalyzes the first committed step in the synthesis of adenosine [26]. Conversely, four DEGs were significantly up-regulated (2.014- to 2.141-fold), e.g., the glutamate synthase large subunit (*B4602_RS02195*) (2.141-fold). Li et al. reported that this enzyme may play a key role in the antimicrobial resistance in cocci through its involvement in folate metabolism or cell membrane integrity [27].

The ribosomes responsible for protein synthesis are one of the main antibiotic targets in bacterial cells [28,29]. The up-regulation of the ribosome metabolic pathway indicates accelerated cell division, which increases the likelihood of gene mutation. In this study, fifteen DEGs were significantly up-regulated (2.003- to 2.791-fold) in *S. aureus* GIM1.441 (*p* < 0.05), e.g., 30S ribosomal protein S8 (*B4602_RS11755*), 50S ribosomal protein L18 (*B4602_RS11745*), and 50S ribosomal protein L5 (*B4602_RS11765*).

Taken together, Fraction 2 of *A. villosum* Lour. altered the eight metabolic pathways in *S. aureus* GIM1.441, thereby likely inhibited the bacterial amino acid and secondary metabolite metabolisms, cell membrane biosynthesis, and resistance. The up-regulated expression of ribosome-related proteins may serve as a self-saving strategy for the bacterium to survive under the unfavorable circumstance elicited by the Fraction 2 treatment.

#### 2.7.2. The Altered Metabolic Pathways in *B. cereus* Y1 Treated with Fraction 2 of *A. villosum* Lour.

**Figure 8:**
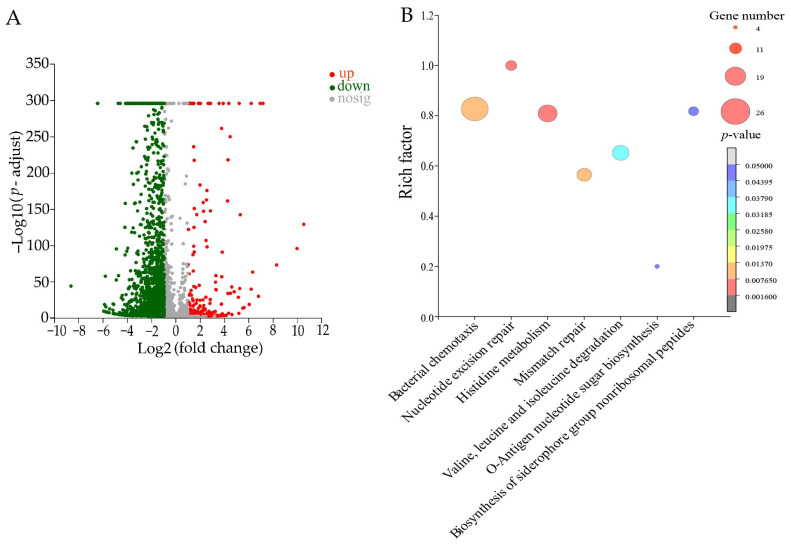
The significantly altered metabolic pathways in *B. cereus* Y1 treated with Fraction 2 of *A. villosum* Lour. (**A**,**B**): Volcano plot of the DGEs and the changed metabolic pathways, respectively.

The DEGs in the treatment group accounted for 44.45% (2363/5316) of the *B. cereus* Y1 genes. Of these, notably, 2173 DEGs were down-regulated (FC ≤ 0.5), whereas 190 DEGs were up-regulated (FC ≥ 2.0). Seven metabolic pathways were significantly altered in *B. cereus* Y1, including bacterial chemotaxis; nucleotide excision repair; histidine metabolism; mismatch repair; valine, leucine, and isoleucine degradation; the biosynthesis of siderophore group nonribosomal peptides; and o-antigen nucleotide sugar biosynthesis (Figure 8, Table S2).

For example, in the bacterial chemotaxis, the expression of 24 DEGs was significantly down-regulated (0.11- to 0.479-fold) at the transcriptional level in *B. cereus* Y1 after being treated with Fraction 2 (*p* < 0.05), e.g., the methyl-accepting chemotaxis protein (MCP) (*EJ379_25705*), chemotaxis signal transduction protein CheV (*EJ379_08390*), flagellar motor protein MotB (*EJ379_23055*), and glutamate O-methyltransferase CheR (*EJ379_05155*). For instance, the MCP plays an important role in cell survival and biodegradation [30], and can also control the direction of flagella motors, promoting cell rolling and smooth swimming [31].

In nucleotide excision repair, ten DEGs were also significantly inhibited (0.18- to 0.431-fold) (*p* < 0.05), e.g., the transcription-repair coupling factor (*EJ379_00305*), DNA polymerase I (*EJ379_23430*), NAD-dependent DNA ligase LigA (*EJ379_01760*), and ATP-dependent helicase (*EJ379_06255*). For example, LigA is crucial for DNA replication and repair in only bacteria and some viruses. LigAs have been reported to be attractive targets for antibacterial drugs [32].

In mismatch repair, the expression of 13 DEGs was significantly down-regulated (0.242- to 0.500-fold) in *B. cereus* Y1 (*p* < 0.05). For example, the DNA mismatch repair endonuclease MutL (*EJ379_19260*) was significantly repressed (0.332-fold). The MutL family of DNA mismatch repair proteins plays a key role in the cleavage and repair of mismatch errors during DNA replication [33].

In valine, leucine, and isoleucine degradation, 12 DEGs were significantly down-regulated (0.179- to 0.49-fold) (*p* < 0.05); in o-antigen nucleotide sugar biosynthesis, 3 DEGs were significantly inhibited (0.073- to 0.018-fold) (*p* < 0.05); and in the biosynthesis of the siderophore group nonribosomal peptides, 9 DEGs were significantly down-regulated as well (0.079- to 0.349-fold) (*p* < 0.05).

Taken together, Fraction 2 of *A. villosum* Lour. altered the seven metabolic pathways in *B. cereus* Y1, thereby hindered the bacterial chemotaxis, flagellar movement, DNA replication and repair, and amino acid metabolisms, leading to its rupture and death.

#### 2.7.3. The Altered Metabolic Pathways in *V. parahemolyticus* B2-28 Treated with Fraction 2 of *A. villosum* Lour.

**Figure 9:**
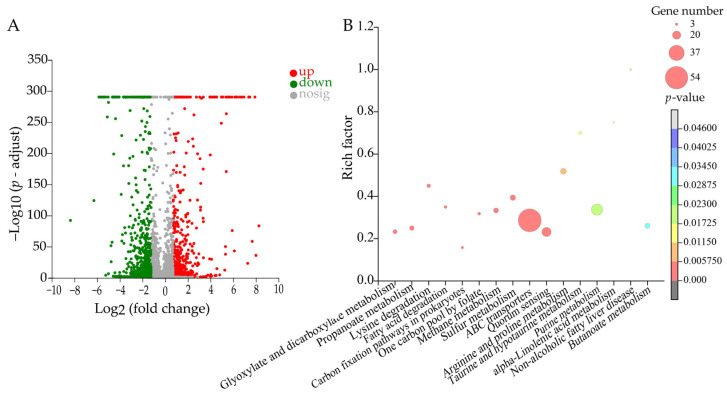
The significantly altered metabolic pathways in *V. parahemolyticus* B2-28 treated with Fraction 2 of *A. villosum* Lour. (**A**,**B**): Volcano plot of the DGEs and the changed metabolic pathways, respectively.

The DEGs in the treatment group accounted for 19.30% (1081/5602) of the *V. parahemolyticus* B2-28 genes. Of these, 648 DEGs were down-regulated (FC ≤ 0.5), whereas 433 DEGs were up-regulated (FC ≥ 2.0). Remarkably, sixteen metabolic pathways were significantly altered in *V. parahemolyticus* B2-28, including glyoxylate and dicarboxylate metabolism; propanoate metabolism; lysine degradation; one carbon pool by folate; fatty acid degradation; methane metabolism; sulfur metabolism; one carbon pool by folate; ABC transporters; QS; arginine and proline metabolism; taurine and hypotaurine metabolism; purine metabolism; alpha-linolenic acid metabolism; non-alcoholic fatty liver disease; and butanoate metabolism (Figure 9, Table S3).

For example, in glyoxylate and dicarboxylate metabolism, the expression of eight DEGs was significantly inhibited (0.126-fold to 0.499-fold) in *V. parahemolyticus* B2-28 after being treated with Fraction 2 of *A. villosum* Lour., whereas two DEGs were significantly up-regulated (2.081- to 4.678-fold) (*p* < 0.05). For instance, the multidrug transporter AcrB (*Vp_B2_28_4316*) was highly repressed (0.126-fold), which is a component of the multidrug efflux pumps that play a key role in the process of bacterial resistance [34]. In contrast, the catalase (*Vp_B2_28_0438*) was significantly up-regulated (2.081-fold), which has been reported to prevent cellular oxidative damage by decomposing hydrogen peroxide into water and oxygen [35].

In propanoate metabolism, the expression of nine DEGs was also significantly inhibited (0.199-fold to 0.432-fold) (*p* < 0.05), e.g., the DNA mismatch repair protein MutT, histone acetyltransferase, and phosphoenolpyruvate protein phosphotransferase (PtsA). The latter was significantly down-regulated (0.264-fold), which is involved in the monosaccharide phosphotransferase system. Nebenzahl et al. [36] reported that the cell-wall-localized PtsA can function as an adhesin, and anti-PtsA antisera were shown to inhibit the adhesion of *S. pneumoniae* to cultured human lung adenocarcinoma cells A549.

In lysine degradation, the expression of six DEGs was significantly inhibited (0.177-fold to 0.482-fold), whereas three DEGs were significantly up-regulated (2.086- to 3.719-fold) in *V. parahemolyticus* B2-28 (*p* < 0.05). For instance, citrate (Si)-synthase (*Vp_B2_28_3274*) was significantly down-regulated (0.453-fold), which catalyzes the initial reaction of the tricarboxylic acid cycle (TCA) [37]. In contrast, the heavy-metal-responsive transcriptional regulator (*Vp_B2_28_3823*) was significantly up-regulated (2.086-fold), which plays a crucial role in metal uptake, isolation, oxidation, or reduction to lower toxicity by regulating the expression of detoxification-related genes [38].

In the ABC transporters, the expression of 43 DEGs was significantly inhibited (0.014-fold to 0.483-fold) and 12 DEGs were significantly up-regulated (2.073- to 27.425-fold) (*p* < 0.05). For example, the oligopeptide ABC transporter ATP-binding protein OppF (*Vp_B2_28_0132*) was significantly down-regulated (0.215-fold), which is essential for spirochete viability in vitro and during infection [39]. Moreover, the serine protease (*Vp_B2_28_0735*) was also significantly inhibited (0.221-fold). The cell-membrane-binding serine protease plays an important role in maintaining cell homeostasis in pathobiology [40].

In the QS, the expression of 20 DEGs was significantly inhibited (0.116-fold to 0.495-fold) in *V. parahemolyticus* B2-28 (*p* < 0.05). Many previous studies have indicated that the formation of QS and biofilm promotes the development of antibiotic resistance in microorganisms [41]. In this study, for example, the DEG encoding a ketol-acid reductoisomerase (KARI) (*Vp_B2_28_4615*) was significantly down-regulated (0.434-fold), which has been reported to be a potential drug target against pathogenic bacteria [42].

In arginine and proline metabolism, the expression of 13 DEGs was significantly inhibited (0.071-fold to 0.284-fold) (*p* < 0.05). In contrast, remarkably, the expression of endonuclease I (*Vp_B2_28_2907*) was strongly enhanced (70.055-fold), suggesting that DNA breakage may have occurred in *V. parahaemolyticus* B2-28 after the Fraction 2 treatment.

In taurine and hypotaurine metabolism, the expression of four DEGs was significantly down-regulated (0.353- to 0.488-fold) in *V. parahaemolyticus* B2-28 (*p* < 0.05). For instance, the RNA chaperone ProQ (*Vp_B2_28_2494*) and tail-specific protease (*Vp_B2_28_2495*) were repressed (0.353-fold and 0.42-fold). The former mediates sRNA-directed gene regulation in Gram-negative bacteria, while the latter is involved in tolerance to heat stress and virulence [43,44]. In addition, the DEG encoding a phage shock protein (Psp) G was greatly down-regulated (0.098-fold). The Psp stress response system can sense and respond to cell membrane damage [45].

Taken together, Fraction 2 of *A. villosum* Lour. significantly altered sixteen metabolic pathways in *V. parahemolyticus* B2-28, and thus hindered the amino acid metabolism, cell membrane biosynthesis, and substance transportation; and repressed the intercellular communication, stress regulation, and virulence, leading to cellular oxidative damage, DNA breakage, and cell death.

Additionally, to validate the transcriptome data, we performed real-time reverse transcription quantitative PCR (RT-qPCR) analysis on fifteen representative DEGs, and the obtained results were generally consistent with the transcriptome data (Tables S4 and S5).

#### 2.7.4. Antibacterial Modes of Fraction 2 of *A. villosum* Lour.

As shown in Table S6, Fraction 2 of *A. villosum* Lour. displayed different antibacterial modes against the Gram-positive and Gram-negative bacteria and hindered a series of metabolic pathways, leading to varying levels of cell damage and even death. On the other hand, the same metabolic pathways, such as o-antigen nucleotide sugar biosynthesis and valine, leucine, and isoleucine metabolisms, were all inhibited in the Gram-positive bacteria *S. aureus* GIM1.441 and *B. cereus* Y1 by Fraction 2. Additionally, some metabolic pathways were only hindered in the Gram-negative bacterium. *parahemolyticus* B2-28 by Fraction 2, such as the repressed substance transporting, intercellular communication, stress regulation, and virulence.

Overall, the results of this study demonstrate that Fraction 2 of *A. villosum* Lour. exerts the strongest inhibitory efficacy on the Gram-negative bacterium *V. parahemolyticus* B2-28, followed by the Gram-positive bacteria *B. cereus* Y1 and *S. aureus* GIM1.441 through multiple antibacterial modes.

### 2.8. Identification of Potential Antibacterial Compounds in Fraction 2 of A. villosum Lour.

Based on the above results, we wondered what compounds functioned in Fraction 2 of *A. villosum* Lour. Therefore, the antibacterial components of Fraction 2 were further identified using the ultra-HPLC and mass spectrometry (UHPLC-MS) technique. As shown in Table 3, eighty-nine compounds were identified. The most abundant compound in Fraction 2 was 4-hydroxyphenylacetylglutamic acid (30.89%), followed by lubiprostone (11.86%), miltirone (10.68%), oleic acid (10.58%), and oxymorphone (5.69%). The other compounds (4.81–0.10%), such as alkaloids, flavonoids, phenols, and coumarins, were also identified (Table 3).

The 4-hydroxyphenylacetylglutamic identified in this study is an acetyl compound of glutamate. It has been reported that this compound can pass through the blood–brain barrier, improve nerve cell metabolism, maintain nervous stress, and reduce blood ammonia [46]. The lubiprostone identified in this study can safely and effectively treat chronic idiopathic constipation and irritable bowel syndrome with constipation [47]. Terpenoids, such as the miltirone identified in this study, have enormous inhibitory potential against microorganisms through different mechanisms such as membrane disruption, anti-QS, and protein and ATP synthesis inhibition [48]. The 8-Geranyloxypsoralen, bergamotine, and 3,4-Dihydrocoumarin identified in this study are coumarins, with pharmacological activities such as antibacterial, anti-inflammatory, and anti-cancer [49]. The piperlonguminine identified in this study is a compound of the alkaloid class that has been proved to have anti-inflammatory activity [50]. The isoquercitrin identified in this study has a variety of chemical protection effects in vitro and in vivo against oxidative stress, cancer, cardiovascular disease, diabetes, and allergic reaction [51].

Taken together, these results have revealed potential antibacterial compounds in Fraction 2 of *A. villosum* Lour., a promising antibacterial source of natural products.

The major limitation of this study was that the top compounds identified in Fraction 2 were not available commercially, therefore, the antibacterial activity of each compound could not be analyzed. It would be interesting to continue with the proposed analysis through a chemical synthesis method to obtain single compounds in future research.

## 3. Materials and Methods

### 3.1. Bacterial Strains and Culture Conditions

The bacterial strains and media used in this study are shown in Table S7. The incubation conditions of the bacterial strains were the same as those described in our recent reports [11,12,13,14].

### 3.2. Extraction of Bacteriostatic Substances from A. villosum Lour.

The fresh fruit samples were purchased from the production base of *A. villosum* Lour. in Yangchun City (22°41′01″ N, 111°16′27″ E), Guangdong Province, China (Figure S2). The fruit is oval, purplish red when ripe, and brown after dry, with a unique and rich aroma. The antibacterial components in the samples were extracted using the M–CE method, as reported in our recent studies [11,12,13,14]. Briefly, the fresh samples were washed with water, cut into small pieces of about 1/4 size, and pre-frozen at −80 °C for 8 h. Thereafter, the samples were further freeze-dried, crushed, and extracted with the methanol–chloroform (2:1, *v*/*v*, analytical grade, Merck KGaA, Darmstadt, Germany) at a solid-to-liquid ratio of 1:10 (*m*/*v*) for 5 h. A certain amount of H_2_O (analytical grade) was added, sonicated, and filtered, and then the MPE and CPE were separated using the same equipment and parameters described in our recent reports [11,12,13,14].

### 3.3. Antibacterial Activity Assay

The sensitivity of the bacterial strains to the MPE and CPE from *A. villosum* Lour. was measured according to the standard method approved by the Clinical and Laboratory Standards Institute, Malvern, PA, USA (CLSI, M100-S23, 2018). The MICs of the extracts from *A. villosum* Lour. were determined against the target strains. The definition of antibacterial activity and MICs were described in our recent reports [11,12,13,14].

### 3.4. Prep-HPLC Analysis

The MPE of *A. villosum* Lour. was isolated using a Waters 2707 autosampler (Waters, Milford, MA, USA) linked with a UPLC Sunfifire C_18_ column (Waters, Milford, MA, USA). The parameters of the Prep-HPLC analysis were the same as those described in our recent reports [11,12,13,14].

### 3.5. UHPLC-MS Analysis.

The UHPLC-MS analysis was carried out by Shanghai Hoogen Biotech, Shanghai, China, using the EXIONLC system (Sciex, Framingham, MA, USA). The running parameters of the UHPLC-MS were the same as those described in our recent reports [11,12,13,14].

### 3.6. Growth Curve Assay

The 1 x MIC and 1/2 x MIC of Fraction 2 of *A. villosum* Lour. were individually added into the bacterial cell culture of the target strains at the mid-logarithmic growth phase (mid-LGP) and then incubated at 37 °C for 24 h. The growth curves of the target strains were determined using the automatic growth curve analyzer (Synergy, BioTekInstruments, Winooski, VT, USA).

### 3.7. SEM Assay

Fraction 2 (1 x MIC) of *A. villosum* Lour. was added into the bacterial cell culture of the target strains and then incubated at 37 °C for 2 h, 4 h, and 6 h, respectively. Bacterial cells were then harvested by centrifugation at 4000 rpm at 4 °C for 10 min, then fixed with 2.50% glutaraldehyde (Shanghai Sangon Biological Engineeing Technology and Service Co., Ltd., Shanghai, China) at 4 °C for 12 h, and dehydrated in the gradient ethanol (30%, 50%, 80%, 90%, and 100%) (Sangon, Shanghai, China) for 15 min, respectively. The samples were observed using the SEM (Hitachi, SU5000, Tokyo, Japan, 5.0 kV, x30,000) [11,12,13,14].

### 3.8. The CSH, CMF, and CMP Assays

The CSH of the target strains was determined according to the method of Cui et al. [52]. Briefly, the target strains at the mid-LGP were treated with Fraction 2 of *A. villosum* Lour for 2 h, 4 h, and 6 h, respectively. A total of 1 mL of hexadecane (National Pharmaceutical Group Corporation Co., Ltd., Shanghai, China) was added to an equal volume of the treated bacterial suspension, mixed for 1 min, and set at 37 °C for 30 min. Thereafter, the absorbance values of the mixture were measured at 600 nm. The CMF of the target strains was determined using the DPH (Sangon, Shanghai, China) as a probe, according to the method described in our recent reports [11,12,13,14]. The CMP of the target strains was measured using the OPNG (Beijing Solarbio Science & Technology Co., Ltd., Beijing, China) as a probe [11,12,13,14].

### 3.9. Bacterial Nucleotide Acid and Protein Exudation Assays

The nucleotide acid exudation of the target strains was measured according to the method of Lin et al. [53], with minor modifications. Briefly, the bacterial cell culture at the mid-LGP was treated with Fraction 2 (1 x MIC) of *A. villosum* Lour for 2 h, 4 h, and 6 h, respectively. The bacterial cell suspension was centrifuged at 4 °C at 3500 rpm for 5 min. Thereafter, the absorbance values of the supernatant were measured at 260 nm.

The bacterial protein exudation was determined according to the method of Atta et al. [54]. Briefly, Fraction 2 (1 x MIC) of *A. villosum* Lour was added into the bacterial cell culture at the mid-LGP and then incubated at a stationary condition at 37 °C for 24 h. The extracellular protein concentrations were determined using the Bradford method protein concentration determination kit (Sangong, Shanghai, China) according to the manufacturer’s instructions.

### 3.10. Illumina RNA Sequencing

The bacterial cell culture at the mid-LGP of the target strains was individually treated with Fraction 2 (1 x MIC) of *A. villosum* Lour for 6 h. The total RNA of the harvested bacterial cells was extracted, purified, and analyzed, as described in our recent reports [11,12,13,14]. Three independently prepared RNA samples were used for each Illumina RNA-sequencing analysis, which was conducted by Shanghai Majorbio Bio-pharm Technology Co., Ltd. (Shanghai, China) using Illumina HiSeq 2500 platform (Illumina, Santiago, CA, USA) [11,12,13,14].

### 3.11. RT-qPCR Assay

The RT-qPCR assay was performed using the same kits and instrument outlined in the method described previously [55]. The oligonucleotide primers (Table S5) were designed using Primier 5.0 software (https://www.premierbiosoft.com (accessed on 28 September 2023), and synthesized by Sangon, Shanghai, China.

### 3.12. Data Analysis

The DEGs were defined, and the altered metabolic pathways were analyzed by referring to the methods described in our recent studies [11,12,13,14]. All tests were conducted in triplicate, and the experimental data were analyzed using the SPSS version 17.0 software (SPSS Inc., Armonk, NY, USA).

## 4. Conclusions

In this study, we first investigated the antibacterial ingredients and modes of the MPE from the fruit of the pharmacophagous plant *A. villosum* Lour. The antibacterial rate of the MPE was 63.60%, targeting 22 species of common pathogenic bacteria. The MPE inhibited 2 species of Gram-positive bacteria, *S. aureus* and *B. cereus*; and 12 species of Gram-negative bacteria, *A. hydrophila*, *P. aeruginosa*, *S. dysenteriae*, *S. flexneri*, *S. sonnei*, *S. enterica* subsp. *enterica* (*ex* Kauffmann and Edwards), *V. alginolyticus*, *V. cholerae*, *V. harveyi*, *V. metschnikovi*, *V. mimicus,* and *V. parahaemolyticus*. The CPE showed an inhibition rate of 54.55% and inhibited one species of Gram-positive bacteria and 11 species of Gram-negative bacteria.

The MPE was further purified by Prep-HPLC, and three different constituents (Fractions 1–3) were obtained. Of these, the Fraction 2 treatment significantly increased the CMF and CMP, reduced the CSH, and damaged the integrity of the cell structure, leading to the leakage of the cellular macromolecules of Gram-positive *S. aureus* GIM1.441 and *B. cereus* Y1 and Gram-negative *V. parahemolyticus* B2-28 (*p* < 0.05). Eighty-nine compounds in Fraction 2 were identified by the UHPLC-MS analysis, among which 4-hydroxyphenylacetylglutamic acid accounted for the highest 30.89%, followed by lubiprostone (11.86%), miltirone (10.68%), and oleic acid (10.58%).

Comparative transcriptomics analysis revealed a series of significantly altered metabolic pathways in the representative Gram-positive and Gram-negative target strains treated with Fraction 2 (*p* < 0.05), indicating multiple antibacterial modes, e.g., hindering the amino acid metabolism and cell membrane biosynthesis, repressing the stress regulation and virulence, and leading to DNA breakage and cell death. Fraction 2 exerted the strongest inhibiting efficiency on Gram-negative *V. parahemolyticus* B2-28, followed by Gram-positive *B. cereus* Y1 and *S. aureus* GIM1.441.

Overall, this study first demonstrates the antibacterial activity of the MPE from the fruit of *A. villosum* Lour. and has provided data for its application in the medicinal and food preservative industries against common pathogens.

## Figures and Tables

**Table 1:** Antibacterial activities of the MPE and CPE from the fruit of *A. villosum* Lour.

Bacterial Strain	DIZ (Diameter, mm)		MIC (μg/mL)
	MPE	CPE	MPE
*Aeromonas hydrophila* ATCC35654	8.00 ± 0.26	10.00 ± 0.53	1024
*Bacillus cereus* Y1	9.50 ± 0.26	8.00 ± 0.56	512
*Enterobacter cloacae* ATCC13047	—	8.00 ± 0.26	—
*Enterobacter cloacae* D1	—	—	—
*Escherichia coli* ATCC8739	—	8.00 ± 0.10	—
*Escherichia coli* K12	—	—	—
*Escherichia coli* ATCC25922	—	9.00 ± 0.36	—
*Enterobacter sakazakii* CMCC45401	—	—	—
*Klebsiella pneumoniae* 7-17-16	—	9.00 ± 0.44	—
*Pseudomonas aeruginosa* ATCC9027	—	8.00 ± 0.17	—
*Pseudomonas aeruginosa* ATCC27853	8.00 ± 0.36	9.00 ± 0.17	1024
*Staphylococcus aureus* GIM1.481	9.50 ± 0.20	—	512
*Staphylococcus aureus* GIM1.441	11.00 ± 0.53	—	128
*Staphylococcus aureus* GIM1.160	—	—	—
*Shigella dysenteriae* CMCC51252	9.00 ± 0.56	—	512
*Salmonella choleraesuis* ATCC13312	—	—	—
*Shigella flexneri* GIM1.238	8.50 ± 0.26	9.00 ± 0.44	1024
*Shigella flexneri* GIM1.539	—	—	—
*Shigella flexneri* GIM1.231	—	—	—
*Salmonella paratyphi* GIM1.235	—	—	—
*Shigella sonnei* GIM1.424	9.00 ± 0.17	—	512
*Shigella sonnei* GIM1.239	—	9.00 ± 0.35	—
*Salmonella enterica* subsp. *enterica* (*ex* Kauffmann and Edwards) Le Minor and Popoff serovar Vellore ATCC15611	9.00 ± 0.17	—	512
*Vibrio cholerae* GIM1.449	8.50 ± 0.26	9.00 ± 0.52	1024
*Vibrio cholerae* B1	—	8.00 ± 0.61	—
*Vibrio parahaemolyticus* B2-28	9.50 ± 0.26	—	256
*Vibrio parahaemolyticus* N2-5	9.00 ± 0.40	—	1024
*Vibrio parahaemolyticus* N9-20	9.00 ± 0.35	—	512
*Vibrio alginolyticus* ATCC33787	8.00 ± 0.50	—	1024
*Vibrio fluvialis* ATCC33809	—	10.00 ± 0.10	—
*Vibrio harveyi* ATCC BAA-1117	8.00 ± 0.53	—	1024
*Vibrio harveyi* ATCC33842	—	10.00 ± 0.26	—
*Vibrio metschnikovi* ATCC700040	9.00 ± 0.30	—	512
*Vibrio mimicus* bio-56759	9.00 ± 0.44	—	512
*Vibrio parahaemolyticus* ATCC17802	9.00 ± 0.50	8.00 ± 0.53	512
*Vibrio parahaemolyticus* ATCC33847	9.00 ± 0.26	10.00 ± 0.44	512
*Vibrio vulnificus* ATCC27562	—	—	—

Note: CPE: chloroform-phase extract. MPE: methanol-phase extract. —: no antibacterial activity. DIZ: diameters of inhibitory zone, including the diameter of the disc (6 mm). MIC: minimum inhibitory concentration. The values are expressed as the mean ± standard deviation (S.D.) of three parallel measurements.

**Table 2:** Antibacterial activities of Fraction 2 of the MPE of *A. villosum Lour*.

Bacterial Strain	DIZ (Diameter, mm)	MIC (μg/mL)
*A. hydrophila* ATCC35654	8.00 ± 0.44	2048
*B. cereus* Y1	11.00 ± 0.00	512
*S. aureus* GIM1.481	9.50 ± 0.15	1024
*S. aureus* GIM1.441	11.50 ± 0.26	256
*S. dysenteriae* CMCC51252	8.00 ± 0.78	2048
*S. flexneri* GIM1.238	8.00 ± 0.61	2048
*S. sonnei* GIM1.424	8.50 ± 0.1	1024
*Salmonella enterica* subsp. *enterica* (*ex* Kauffmann and Edwards) Le Minor and Popoff serovar Vellore ATCC15611	8.00 ± 0.35	2048
*V. parahemolyticus* B2-28	11.00 ± 0.26	512
*V. parahemolyticus* N2-5	8.00 ± 0.61	2048
*V. parahemolyticus* N9-20	7.00 ± 0.10	4096
*V. alginolyticus* ATCC33787	8.00 ± 0.36	2048
*V. mimicus* bio-56759	9.00 ± 0.56	1024
*V. parahemolyticus* ATCC17802	10.00 ± 0.26	1024
*V. parahemolyticus* ATCC33847	8.00 ± 0.46	2048

**Table 3:** Potential antibacterial compounds identified in Fraction 2 of *A. villosum* Lour. by the UHPLC-MS analysis.

Identified Compound	Rt (min)	Compound Nature	Formula	Exact Mass	Fraction Area (%)
4-Hydroxyphenylacetylglutamic acid	12.99	Glutamate and derivatives	C_13_H_15_NO_6_	281.09	30.89%
Lubiprostone	12.75	Fatty acyls	C_20_H_32_F_2_O_5_	390.22	11.86%
Miltirone	12.98	Diterpenoids	C_19_H_22_O_2_	282.16	10.68%
Oleic acid	13.03	Fatty acyls	C_18_H_34_O_2_	282.26	10.58%
Oxymorphone	11.18	Morphinane and derivatives	C_17_H_19_NO_4_	301.13	5.69%
Piperlonguminine	10.57	Alkaloids	C_16_H_19_NO_3_	273.14	4.81%
Adenosine	2.58	Amino acid and derivatives	C_10_H_13_N_5_O_4_	267.1	3.74%
Nandrolone	10.58	Steroids and steroid derivatives	C_18_H_26_O_2_	274.19	2.89%
Palmitic acid	12.92	Lipids	C_16_H_32_O_2_	256.24	2.64%
Sarracine	13.14	Alkaloids	C_18_H_27_NO_5_	337.19	2.27%
Glucose 1-phosphate	13.00	Organo-oxygen compounds	C_6_H_13_O_9_P	260.03	2.23%
Artemisinin	13.02	Sesquiterpenoids	C_15_H_22_O_5_	282.15	1.83%
Erucic acid	13.28	Fatty acyls	C_22_H_42_O_2_	338.32	0.93%
2-Phenylacetamide	2.52	Benzene and substituted derivatives	C_8_H_9_NO	135.07	0.92%
Crotonoside	2.40	Alkaloids	C_10_H_13_N_5_O_5_	283.09	0.76%
22-Dehydroclerosterol	12.59	Steroids	C_29_H_46_O	410.35	0.72%
Guanine	2.63	Nucleotide and derivates	C_5_H_5_N_5_O	151.05	0.64%
Wighteone	13.01	Flavonoids	C_20_H_18_O_5_	338.12	0.52%
4-Hydroxybenzaldehyde	1.91	Phenols	C_7_H_6_O_2_	122.04	0.47%
Octadecanamide	13.02	Fatty acyls	C_18_H_37_NO	283.29	0.47%
8-Geranyloxypsoralen	13.29	Coumarins	C_21_H_22_O_4_	338.15	0.45%
Kirenol	13.03	Diterpenoids	C_20_H_34_O_4_	338.25	0.34%
7-(4-Hydroxyphenyl)-1-phenyl-4-hepten-3-one	12.53	Phenols	C_19_H_20_O_2_	280.15	0.32%
Glycerophosphocholine	12.87	Cholines	C_8_H_20_NO_6_P	257.22	0.30%
Supinine	12.99	Alkaloids	C_15_H_25_NO_4_	283.18	0.27%
Octyl gallate	12.97	Phenols	C_15_H_22_O_5_	282.15	0.22%
Inosine	2.60	Nucleotide and derivates	C_10_H_12_N_4_O_5_	268.08	0.21%
Moracin C	13.99	Phenols	C_19_H_18_O_4_	310.12	0.20%
AICA-riboside	13.28	Imidazole ribonucleosides and ribonucleotides	C_9_H_15_N_4_O_8_P	338.06	0.19%
Glucose 1-phosphate	13.03	Organo-oxygen compounds	C_6_H_13_O_9_P	260.03	0.14%
Kirenol	13.16	Diterpenoids	C_20_H_34_O_4_	338.25	0.12%
Isoquercitrin	6.06	Flavonoids	C_21_H_20_O_12_	464.1	0.10%
Oleamide	12.54	Fatty acyls	C_18_H_35_NO	281.27	0.10%
DL-Tyrosine	1.90	Monophenols amino acids	C_9_H_11_NO_3_	181.07	0.08%
L-Pipecolic acid	0.69	Amino acid and derivatives	C_6_H_11_NO_2_	129.08	0.08%
Stearic acid	13.02	Fatty acyls	C_18_H_36_O_2_	284.27	0.07%
Panaxynol	12.57	Miscellaneous	C_17_H_24_O	244.18	0.06%
Xanthosine	2.61	Nucleotide and derivates	C_10_H_12_N_4_O_6_	284.08	0.05%
N1-Methyl-4-pyridone-3-carboxamide	2.66	Pyridines and derivatives	C_7_H_8_N_2_O_2_	152.06	0.05%
2-(2-Hydroxy-2-propyl)-5-methyl-5-vinyltetrahydrofuran	5.47	Monoterpenoids	C_10_H_18_O_2_	170.13	0.05%
α-Isopropylmalate	10.79	Nucleotide and derivates	C_7_H_12_O_5_	176.07	0.04%
Isoquercitrin	6.22	Flavonoids	C_21_H_20_O_12_	464.1	0.04%
Bergamotine	13.03	Coumarins	C_21_H_22_O_4_	338.15	0.04%
Seneciphylline	9.06	Alkaloids	C_18_H_23_NO_5_	333.16	0.03%
Cianidanol	4.50	Flavonoids	C_15_H_14_O_6_	290.08	0.03%
3,4,5-Trimethoxycinnamyl alcohol	10.40	Phenylpropanoids	C_12_H_16_O_4_	224.1	0.03%
Procyanidin B2	4.78	Flavonoids	C_30_H_26_O_12_	578.14	0.03%
(Z)-Aconitic acid	1.46	Organic acids and derivatives	C_6_H_6_O_6_	174.02	0.03%
Astragalin	6.52	Flavonoids	C_21_H_20_O_11_	448.1	0.03%
Kazinol A	13.26	Phenols	C_25_H_30_O_4_	394.21	0.02%
Epifriedelanol	12.29	Terpenoids	C_30_H_52_O	428.4	0.02%
Stigmasterol	13.12	Steroids	C_29_H_48_O	412.37	0.02%
4-Propylphenol	10.56	Benzene and substituted derivatives	C_9_H_12_O	136.09	0.02%
Kaempferol-3-O-glucorhamnoside	6.29	Flavonoids	C_27_H_30_O_15_	594.16	0.02%
Geranyl acetate	9.58	Monoterpenoids	C_12_ H_20_O_2_	196.15	0.01%
Tiliroside	7.92	Flavonoids	C_30_H_26_O_13_	594.14	0.01%
3,4-Dihydrocoumarin	6.68	Coumarins	C_9_H_8_O_2_	148.05	0.01%
20-HETE	13.28	Fatty acyls	C_20_H_32_O_3_	320.24	0.01%
Herniarin	7.69	Coumarins	C_10_H_8_O_3_	176.05	0.01%
Fingolimod hydrochloride	13.11	Sphingosine analogues	C_19_H_34_C_1_NO_2_	343.93	0.01%
4-Hydroxybenzoic acid	1.08	Phenols	C_7_H_6_O_3_	138.03	0.01%
D-Glucuronic acid lactone	4.22	Ketones	C_6_H_8_O_6_	176.03	0.01%
Tricetin	8.05	Flavonoids	C_15_H_10_O_7_	302.04	0.01%
Benzocaine	3.05	Benzene and substituted derivatives	C_9_H_11_NO_2_	165.08	0.01%
Taxiphyllin	13.33	Phenols	C_14_H_17_NO_7_	311.1	0.01%
Thymol	10.86	Phenols	C_10_H_14_O	150.1	0.01%
5-Aminovaleric acid	1.11	Amino acid and derivatives	C_5_H_11_NO_2_	117.08	0.01%
3,5,7-Trimethoxyflavone	10.8	Flavonoids	C_18_H_16_O_5_	312.1	0.01%
N-Methyl-4-pyridone-3-carboxamide	2.63	Pyridines and derivatives	C_7_H_8_N_2_O_2_	152.06	0.01%
9,10-DiHOME	13.3	Linoleic acid diol derivative	C_18_H_34_O_4_	314.25	0.01%
Physalin O	12.39	Steroids and steroid derivatives	C_28_H_32_O_10_	528.2	0.01%
Azelaic acid	6.81	Organic acids	C_9_H_16_O_4_	188.1	0.01%
3-Indolebutyric acid	9.00	Alkaloids	C_12_H_13_NO_2_	203.09	0.01%
Rutin	5.85	Flavonoids	C_27_H_30_O_16_	610.15	0.01%
Niazirin	12.54	Saccharides	C_14_H_17_NO_5_	279.11	0.01%
1-Octacosanol	13.20	Fatty alcohol	C_28_H_58_O	410.45	0.01%
Limonexic acid	12.55	Triterpenoids	C_26_H_30_O_10_	502.18	0.01%
Taraxasterone	13.85	Triterpenoids	C_30_H_48_O	424.37	0.01%
Mitraphylline	13.02	Alkaloids	C_21_H_24_N_2_O_4_	368.17	0.01%
DL-α-Tocopherol acetate	13.37	Vitamin E derivatives	C_31_H_52_O_3_	472.39	0.01%
Trans-Cinnamaldehyde	6.34	Phenylpropanoids	C_9_H_8_O	132.06	0.01%
Caffeoyl alcohol	10.09	Phenols	C_9_H_10_O_3_	166.06	0.01%
Calycosin	9.10	Flavonoids	C_16_H_12_O_5_	284.07	0.01%
Biochanin A	11.38	Flavonoids	C_16_H_12_O_5_	284.07	0.01%
Nicotiflorin	12.34	Flavonoids	C_27_H_30_O_15_	594.16	0.01%
Tiliroside	8.17	Flavonoids	C_30_H_26_O_13_	594.14	0.01%
1-Isomangostin	13.16	Xanthones	C_24_H_26_O_6_	410.17	0.01%
Sterebin F	13.03	Terpenoids	C_20_H_34_O_4_	338.25	0.01%
6,8-Diprenylnaringenin	12.62	Flavonoids	C_25_H_28_O_5_	408.19	0.01%

## Data Availability

Data are contained within the article and Supplementary Materials. The complete lists of the DEGs in the three target strains are available in the NCBI SRA database (https://sub-mit.ncbi.nlm.nih.gov/subs/bioproject/, accessed on 25 September 2023) under the accession number PRJNA1020669.

## Supplementary Materials

The following supporting information can be downloaded at: https://www.mdpi.com/article/10.3390/plants13060834/s1, Table S1: The major altered metabolic pathways in *S. aureus* GIM1.441 treated with Fraction 2 of *A. villosum* Lour.; Table S2: The major altered metabolic pathways in *B. cereus* Y1 treated with Fraction 2 of *A. villosum* Lour.; Table S3: The major altered metabolic pathways in *V. parahemolyticus* B2-28 treated with Fraction 2 of *A. villosum* Lour.; Table S4: Comparison of the major altered metabolic pathways in the three target strains treated with Fraction 2 of *A. villosum* Lour.; Table S5: The oligonucleotide primers designed and used in the RT-qPCR assay; Table S6: The relative expression of the representative DEGs by the RT-qPCR assay; Table S7: The bacterial strains and media used in this study; Figure S1: Growth curves of the target strains treated with Fraction 2 of *A. villosum* Lour. (A–C): *S. aureus* GIM1.441, *B. cereus* Y1, and *V. parahemolyticus* B2-28, respectively. The strains were incubated in the TSB medium at 37 °C; Figure S2. The plant of *A. villosum* Lour. (A–C): the different plant growth periods.
