# Supplementary material for: Antibacterial Ingredients and Modes of the Methanol-Phase Extract from the Fruit of Amomum villosum Lour
Source: Plants (Basel). 2024 Mar 14;13(6):834. doi: 10.3390/plants13060834 (PMC10975419; doi:10.3390/plants13060834)
Supplement: Supplementary file 1 [file plants-13-00834-s001.zip › plants-2902650-supplementary.pdf]

plants MDPI

Supplementary Materials

Table S1. The major altered metabolic pathways in *S. aureus* GIM1.441 treated by Fraction 2 of *A. villosum* Lour.

Metabolic Pathway	Gene ID	Fold Change	Gene Description
Valine, leucine and isoleucine biosynthesis	*B4602_RS11645*	4.692	Acetolactate synthase AlsS
	B4602_RS10775	0.447	Ketol-acid reductoisomerase
	B4602_RS10780	0.390	2-isopropylmalate synthase
	B4602_RS10800	0.394	Threonine ammonia-lyase IlvA
	B4602_RS07310	2.450	Bifunctional threonine ammonia-lyase/L-serine ammonia-lyase TdcB
Biosynthesis of various other secondary metabolites	*B4602_RS00415*	2.968	Bifunctional transcriptional regulator/O-phospho-L-serine synthase SbnI
	B4602_RS00400	3.046	3-(L-alanin-3-ylcarbamoyl)-2-[(2-aminoethylcarbamoyl)methyl]-2 -hydroxypropanoate synthase SbnF
	B4602_RS00395	4.626	L-2,3-diaminopropanoate--citrate ligase SbnE
	B4602_RS00385	2.880	Staphyloferrin B biosynthesis protein SbnC
	B4602_RS00410	2.571	Staphyloferrin B biosynthesis decarboxylase SbnH
Ascorbate and aldarate metabolism	*B4602_RS11165*	2.531	Aldehyde dehydrogenase family protein
	B4602_RS01530	8.846	PTS ascorbate transporter subunit IIC
	B4602_RS01540	7.963	PTS sugar transporter subunit IIA
	B4602_RS01535	6.872	PTS sugar transporter subunit IIB
Ribosome	*B4602_RS11755*	2.573	30S ribosomal protein S8
	B4602_RS11745	2.164	50S ribosomal protein L18
	B4602_RS11705	2.103	30S ribosomal protein S13
	B4602_RS11765	2.497	50S ribosomal protein L5
	B4602_RS11800	2.003	50S ribosomal protein L22
	B4602_RS11790	2.094	50S ribosomal protein L16
	B4602_RS11770	2.500	50S ribosomal protein L24
	B4602_RS11750	2.403	50S ribosomal protein L6
	B4602_RS11775	2.199	50S ribosomal protein L14
	B4602_RS11760	2.791	Type Z 30S ribosomal protein S14
	B4602_RS11805	2.116	30S ribosomal protein S19
	B4602_RS11780	2.240	30S ribosomal protein S17
	B4602_RS11735	2.008	50S ribosomal protein L30
	B4602_RS11710	2.304	50S ribosomal protein L36
	B4602_RS11785	2.067	50S ribosomal protein L29

C5-Branched dibasic acid metabolism	*B4602_RS11640*	4.678	Acetolactate decarboxylase
	B4602_RS10785	0.379	3-isopropylmalate dehydrogenase
	B4602_RS10790	0.414	3-isopropylmalate dehydratase large subunit
	B4602_RS10795	0.303	3-isopropylmalate dehydratase small subunit
Alanine, aspartate and glutamate metabolism	*B4602_RS04620*	2.062	Argininosuccinate lyase
	B4602_RS02195	2.141	Glutamate synthase large subunit
	B4602_RS00095	0.413	Adenylosuccinate synthase
	B4602_RS02200	2.014	Glutamate synthase subunit beta
	B4602_RS05210	0.472	Amidophosphoribosyltransferase
	B4602_RS07315	2.042	Alanine dehydrogenase
O-Antigen nucleotide sugar biosynthesis	*B4602_RS00445*	0.214	NAD-dependent epimerase/dehydratase family protein
	B4602_RS00575	4.175	Type 8 capsular polysaccharide synthesis protein Cap8F
	B4602_RS00570	4.080	Type 8 capsular polysaccharide synthesis protein Cap8E
	B4602_RS00580	3.171	Type 8 capsular polysaccharide synthesis protein Cap8G
Biosynthesis of various antibiotics	*B4602_RS00375*	8.641	2,3-diaminopropionate biosynthesis protein SbnA
	B4602_RS00380	3.155	N-[(2S)-2-amino-2-carboxyethyl]-L-glutamate dehydrogenase SbnB

Table S2. The major altered metabolic pathways in *B. cereus* Y1 treated by Fraction 2 of *A. villosum* Lour.

**Metabolic Pathway**	**Gene ID**	**Fold Change**	**Gene Description**
Bacterial chemotaxis	*EJ379_25705*	0.136	Methyl-accepting chemotaxis protein
	EJ379_08390	0.110	Chemotaxis signal transduction protein CheV
	EJ379_08260	0.199	Chemotaxis protein CheA
	EJ379_23055	0.148	Flagellar motor protein MotB
	EJ379_05160	0.311	Response regulator
	EJ379_02860	0.101	HAMP domain-containing protein
	EJ379_02170	0.188	Methyl-accepting chemotaxis protein
	EJ379_10215	0.120	HAMP domain-containing protein
	EJ379_25580	0.259	Methyl-accepting chemotaxis protein
	EJ379_23060	0.162	Flagellar motor protein MotA
	EJ379_08265	0.267	Flagellar motor switch phosphatase FliY
	EJ379_08255	0.261	Response regulator
	EJ379_02085	0.188	Methyl-accepting chemotaxis protein
	EJ379_05155	0.312	Protein-glutamate O-methyltransferase CheR
	EJ379_08345	0.217	Flagellar motor switch protein FliG
	EJ379_03465	0.358	Methyl-accepting chemotaxis protein
	EJ379_08280	0.369	Protein-glutamate O-methyltransferase CheR
	EJ379_08435	0.196	Flagellar motor switch protein FliM

	EJ379_25710	0.108	Methyl-accepting chemotaxis protein
	EJ379_08245	0.243	Flagellar motor protein MotP
	EJ379_08250	0.463	Flagellar motor protein MotS
	EJ379_05760	0.175	Methyl-accepting chemotaxis protein
	EJ379_17320	0.406	Methyl-accepting chemotaxis protein
	EJ379_08430	0.479	Flagellar motor switch protein FliN
Nucleotide excision repair	*EJ379_00305*	0.180	Transcription-repair coupling factor
	EJ379_26385	0.265	Excinuclease ABC subunit UvrA
	EJ379_23430	0.415	DNA polymerase I
	EJ379_23095	0.241	Excinuclease ABC subunit C
	EJ379_01760	0.260	NAD-dependent DNA ligase LigA
	EJ379_26390	0.431	Excinuclease ABC subunit B
	EJ379_01755	0.380	DNA helicase PcrA
	EJ379_06255	0.410	ATP-dependent helicase
	EJ379_05330	0.330	Helicase
	EJ379_14385	0.280	DNA helicase UvrD
Histidine metabolism	*EJ379_18655*	0.382	Formimidoylglutamase
	EJ379_18175	0.410	Aldehyde dehydrogenase DhaS
	EJ379_18660	0.409	Imidazolonepropionase
	EJ379_18665	0.353	Urocanate hydratase
	EJ379_18670	0.486	Histidine ammonia-lyase
	EJ379_07680	0.491	Histidinol-phosphate transaminase
	EJ379_07135	0.265	Histidinol dehydrogenase
	EJ379_06560	0.398	Aldehyde dehydrogenase
	EJ379_07125	0.307	ATP phosphoribosyltransferase regulatory subunit
	EJ379_07170	0.220	Histidinol phosphate phosphatase domain-containing protein
	EJ379_07150	0.056	1-(5-phosphoribosyl)-5-((5-phosphoribosylamino)methylideneamino)imidazole 2-4-carboxamide isomerase
	EJ379_07130	0.185	ATP phosphoribosyltransferase
	EJ379_07145	0.089	Imidazole glycerol phosphate synthase subunit HisH
	EJ379_07140	0.263	Imidazoleglycerol-phosphate dehydratase HisB
	EJ379_15060	2.156	Histidinol-phosphate transaminase
	EJ379_07160	0.036	Phosphoribosyl-AMP cyclohydrolase
	EJ379_07155	0.068	Imidazoleglycerol phosphate synthase cyclase subunit
Mismatch repair	*EJ379_27960*	0.382	Single-stranded DNA-binding protein
	EJ379_23520	0.263	DNA polymerase III subunit alpha
	EJ379_19260	0.332	DNA mismatch repair endonuclease MutL
	EJ379_19265	0.421	DNA mismatch repair protein MutS
	EJ379_00130	0.480	DNA polymerase III subunit gamma/tau

	EJ379_21375	0.332	Exodeoxyribonuclease VII large subunit
	EJ379_00010	0.242	DNA polymerase III subunit beta
	EJ379_19505	0.451	PolC-type DNA polymerase III
	EJ379_23250	0.391	Endonuclease MutS2
	EJ379_18460	0.462	3'-5' exonuclease
	EJ379_13795	0.388	DNA polymerase III subunit beta
	EJ379_21370	0.267	Exodeoxyribonuclease VII small subunit
	EJ379_17790	0.500	Endonuclease MutS2
Valine, leucine and isoleucine degradation	*EJ379_21270*	0.198	3-methyl-2-oxobutanoate dehydrogenase subunit beta
	EJ379_21275	0.188	3-methyl-2-oxobutanoate dehydrogenase subunit alpha
	EJ379_21280	0.180	Dihydrolipoyl dehydrogenase
	EJ379_21290	0.286	Leucine dehydrogenase
	EJ379_20560	0.413	Acetyl-CoA C-acetyltransferase
	EJ379_21265	0.179	2-oxo acid dehydrogenase subunit E2
	EJ379_20285	0.315	Dihydrolipoyl dehydrogenase
	EJ379_11710	0.490	CoA-acylating methylmalonate-semialdehyde dehydrogenase
	EJ379_12715	0.434	Hydroxymethylglutaryl-CoA lyase
	EJ379_27290	0.409	Acyl-CoA dehydrogenase AcdA
	EJ379_07085	0.319	Branched-chain-amino-acid transaminase
	EJ379_20660	0.484	NAD(P)-dependent oxidoreductase
	EJ379_01835	4.544	4-aminobutyrate-2-oxoglutarate transaminase
	EJ379_18540	4.023	Acetyl-CoA C-acyltransferase
O-Antigen nucleotide sugar biosynthesis	*EJ379_27830*	2.904	UDP-glucose 4-epimerase GalE
	EJ379_02515	0.118	SDR family NAD(P)-dependent oxidoreductase
	EJ379_26945	0.073	UTP-glucose-1-phosphate uridylyltransferase GalU
	EJ379_01610	0.118	NAD-dependent epimerase/dehydratase family protein
Biosynthesis of siderophore group nonribosomal peptides	*EJ379_11790*	0.338	Non-ribosomal peptide synthetase
	EJ379_11780	0.247	(2,3-dihydroxybenzoyl)adenylate synthase
	EJ379_10065	0.293	IucA/IucC family siderophore biosynthesis protein
	EJ379_11775	0.228	Isochorismate synthase DhbC
	EJ379_10070	0.274	Siderophore biosynthesis protein
	EJ379_11785	0.327	Isochorismatase family protein
	EJ379_10075	0.349	Acyl-CoA synthetase
	EJ379_10085	0.270	Petrobactin biosynthesis protein AsbE
	EJ379_10090	0.079	Sugar phosphate isomerase/epimerase

Table S3. The major altered metabolic pathways in *V. parahemolyticus* B2-28 treated by Fraction 2 of *A. villosum* Lour.

Metabolic Pathway	Gene ID	Fold Change	Gene Description
Glyoxylate and dicarboxylate metabolism	*Vp_B2_28_3885*	4.678	Hypothetical protein
	Vp_B2_28_3268	0.288	Membrane protein
	Vp_B2_28_4325	0.214	Transcriptional regulator
	Vp_B2_28_4324	0.253	Glycine cleavage system aminomethyltransferase T
	Vp_B2_28_4323	0.350	Glycine cleavage system aminomethyltransferase T
	Vp_B2_28_2435	0.466	tRNA guanosine(34) transglycosylase Tgt
	Vp_B2_28_2434	0.499	Preprotein translocase subunit YajC
	Vp_B2_28_1968	0.184	Preprotein translocase subunit SecD
	Vp_B2_28_4316	0.126	Multidrug transporter AcrB
	Vp_B2_28_0438	2.081	Catalase
Propanoate metabolism	*Vp_B2_28_2170*	0.271	Trypsin
	Vp_B2_28_2168	0.432	DNA mismatch repair protein MutT
	Vp_B2_28_5268	4.475	FMN-dependent NADH-azoreductase
	Vp_B2_28_5269	7.930	RNA helicase
	Vp_B2_28_2171	0.325	Histone acetyltransferase
	Vp_B2_28_5619	0.264	Phosphoenolpyruvate-protein phosphotransferase
	Vp_B2_28_2169	0.199	DNA mismatch repair protein MutT
	Vp_B2_28_2172	0.369	Histone acetyltransferase
	Vp_B2_28_2173	0.297	Propionyl-CoA synthetase
	Vp_B2_28_5544	0.350	Hypothetical protein
	Vp_B2_28_5206	0.279	Formate dehydrogenase accessory protein FdhD
Lysine degradation	*Vp_B2_28_3274*	0.453	Citrate (Si)-synthase
	Vp_B2_28_3823	2.086	Heavy metal-responsive transcriptional regulator
	Vp_B2_28_1176	0.482	Hypothetical protein
	Vp_B2_28_3822	2.616	Hypothetical protein
	Vp_B2_28_2037	0.216	Agmatine deiminase
	Vp_B2_28_2035	0.216	N-carbamoylputrescine amidase
	Vp_B2_28_2034	0.177	Gamma-glutamylputrescine oxidoreductase
	Vp_B2_28_2033	0.145	Gamma-glutamylputrescine oxidoreductase
	Vp_B2_28_1175	3.719	Cystatin domain protein
Fatty acid degradation	*Vp_B2_28_4879*	0.256	NAD(P)-dependent oxidoreductase
	Vp_B2_28_2279	0.180	Chemotaxis protein
	Vp_B2_28_0250	0.344	3-ketoacyl-CoA thiolase
	Vp_B2_28_0998	0.303	Acyl-CoA dehydrogenase
	Vp_B2_28_0996	0.326	Acyl-CoA dehydrogenase
	Vp_B2_28_0966	0.451	Long-chain-fatty-acid-CoA ligase
	Vp_B2_28_0997	0.174	Acyl-CoA dehydrogenase
Carbon fixation pathways in prokaryotes	*Vp_B2_28_3275*	0.374	Succinate dehydrogenase, cytochrome b556 subunit
	Vp_B2_28_0565	0.383	Aconitate hydratase B

	Vp_B2_28_5208	0.041	Hexose phosphate transporter
	Vp_B2_28_3538	0.491	LysR family transcriptional regulator
	Vp_B2_28_0656	0.453	Mannitol repressor family protein
	Vp_B2_28_2724	0.310	Dnd system-associated protein 3
One carbon pool by folate	*Vp_B2_28_3815*	0.245	Hypothetical protein
	Vp_B2_28_4318	0.097	Multidrug transporter AcrB
	Vp_B2_28_4317	0.149	Multidrug transporter AcrB
	Vp_B2_28_2725	0.406	Hypothetical protein
	Vp_B2_28_3454	0.479	Fatty acid hydroxylase superfamily protein
	Vp_B2_28_2764	2.500	Met repressor
	Vp_B2_28_4138	0.080	Ligase
Methane metabolism	*Vp_B2_28_1542*	0.369	Universal stress protein
	Vp_B2_28_4321	0.257	TetR family transcriptional regulator
	Vp_B2_28_4320	0.099	Hypothetical protein
	Vp_B2_28_3883	4.586	Formate dehydrogenase
	Vp_B2_28_5173	2.388	Nitrate transporter system, permease component
	Vp_B2_28_0682	2.134	Formate dehydrogenase
	Vp_B2_28_5174	2.659	Nitrate ABC transporter ATP-binding protein
	Vp_B2_28_2596	2.270	Hypothetical protein
	Vp_B2_28_3884	4.607	Hypothetical protein
	Vp_B2_28_0681	2.262	Formate dehydrogenase
	Vp_B2_28_0680	2.430	Formate dehydrogenase
	Vp_B2_28_0679	3.703	Formate dehydrogenase subunit alpha
Sulfur metabolism	*Vp_B2_28_2813*	0.088	Iron-hydroxamate ABC transporter substrate-binding protein
	Vp_B2_28_2811	0.092	tRNA dihydrouridine synthase DusA
	Vp_B2_28_2812	0.098	Phage shock protein G
	Vp_B2_28_2814	0.110	Hypothetical protein
	Vp_B2_28_4010	0.196	Hypothetical protein
	Vp_B2_28_4114	4.401	Exonuclease
	Vp_B2_28_5645	4.972	Dimethylsulfoxide reductase subunit B
	Vp_B2_28_2815	0.080	Sulfite reductase
	Vp_B2_28_4113	3.789	Sensor domain-containing diguanylate cyclase
	Vp_B2_28_5675	3.883	3-hydroxyisobutyrate dehydrogenase
	Vp_B2_28_2059	3.905	Chemotaxis protein CheY
	Vp_B2_28_4870	2.491	Prevent-host-death family protein
	Vp_B2_28_3206	2.021	Amino acid acetyltransferase
ABC transporters	*Vp_B2_28_0738*	0.159	Amino acid ABC transporter periplasmic amino acid-binding protein
	Vp_B2_28_0132	0.215	Oligopeptide ABC transporter ATP-binding protein OppF
	Vp_B2_28_0133	0.236	Oligopeptide ABC transporter ATP-binding protein OppD
	Vp_B2_28_0134	0.176	Peptide ABC transporter permease

Vp_B2_28_0135	0.178	Peptide ABC transporter permease
Vp_B2_28_0136	0.306	Peptide ABC transporter substrate-binding protein
Vp_B2_28_0735	0.221	Serine protease
Vp_B2_28_0737	0.201	Amino acid transporter
Vp_B2_28_3870	5.298	Hypothetical protein
Vp_B2_28_5552	0.022	Choline ABC transporter substrate-binding protein
Vp_B2_28_5551	0.020	Methylmalonate-semialdehyde dehydrogenase (acylating)
Vp_B2_28_3093	0.052	DNA polymerase III subunit epsilon
Vp_B2_28_3096	0.062	Cyclic nucleotide-binding protein
Vp_B2_28_1128	27.425	RND transporter
Vp_B2_28_3097	0.057	Cyclic nucleotide-binding protein
Vp_B2_28_0062	0.364	ABC transporter substrate-binding protein
Vp_B2_28_3094	0.079	Cyclic nucleotide-binding protein
Vp_B2_28_0739	0.195	ABC transporter
Vp_B2_28_5553	0.014	Hypothetical protein
Vp_B2_28_4436	2.295	LysR family transcriptional regulator
Vp_B2_28_3393	0.225	Hypothetical protein
Vp_B2_28_3391	0.383	Orotate phosphoribosyltransferase
Vp_B2_28_3392	0.265	Ribonuclease PH
Vp_B2_28_3868	2.087	Flagellar synthesis chaperone protein
Vp_B2_28_3732	0.387	Thiol:disulfide interchange protein DsbE
Vp_B2_28_0736	0.193	Histidine/lysine/arginine/ornithine ABC transporter ATP -binding protein HisP
Vp_B2_28_4437	2.073	Cytochrome biogenesis protein
Vp_B2_28_2754	0.345	Iron transporter FeoA
Vp_B2_28_3730	0.483	C-type cytochrome biogenesis protein CcmI
Vp_B2_28_4300	0.343	ABC transporter permease
Vp_B2_28_4094	0.172	Hypothetical protein
Vp_B2_28_4301	0.299	Hypothetical protein
Vp_B2_28_3869	2.966	N-acetylglucosamine kinase
Vp_B2_28_4439	2.332	Universal stress protein UspA
Vp_B2_28_4386	2.479	LysR family transcriptional regulator
Vp_B2_28_3731	0.370	Cystathionine gamma-synthase
Vp_B2_28_4093	0.263	Alcohol dehydrogenase
Vp_B2_28_4499	2.963	FAD-linked oxidase
Vp_B2_28_4303	0.181	Collagenase
Vp_B2_28_4498	2.356	Arginine ABC transporter substrate-binding protein
Vp_B2_28_4385	2.495	Methyltransferase
Vp_B2_28_3639	0.219	Electron transporter RnfB
Vp_B2_28_2755	0.296	Hypothetical protein
Vp_B2_28_2575	4.648	Hypothetical protein
Vp_B2_28_4594	0.162	Thiaminase II

	Vp_B2_28_4169	0.430	Hypothetical protein
	Vp_B2_28_5464	0.196	HNH endonuclease
	Vp_B2_28_5432	0.435	GGDEF domain-containing protein
	Vp_B2_28_2324	0.366	Arylsulfatase
	Vp_B2_28_0378	0.294	Phosphate ABC transporter, permease protein PstA
	Vp_B2_28_5366	0.103	TetR family transcriptional regulator
	Vp_B2_28_5602	0.066	Phosphoribosylglycinamide formyltransferase
	Vp_B2_28_2576	0.380	PreQ(1) synthase
	Vp_B2_28_3965	0.426	Hypothetical protein
	Vp_B2_28_4833	0.465	Glyoxalase
Quorum sensing	*Vp_B2_28_1994*	0.149	Membrane protein
	Vp_B2_28_0547	0.116	Peptide ABC transporter substrate-binding protein
	Vp_B2_28_4613	0.429	Peptide ABC transporter, periplasmic peptide-binding protein
	Vp_B2_28_0587	0.282	LuxR family transcriptional regulator
	Vp_B2_28_4612	0.448	ABC transporter ATP-binding protein
	Vp_B2_28_4614	0.315	Transcriptional regulator IlvY
	Vp_B2_28_4615	0.434	Ketol-acid reductoisomerase
	Vp_B2_28_1993	0.154	Carotenoid dehydrogenase
	Vp_B2_28_0549	0.140	Peptide ABC transporter permease
	Vp_B2_28_3425	2.705	LPS biosynthesis protein
	Vp_B2_28_1951	0.495	Conserved hypothetical protein
	Vp_B2_28_0548	0.191	Peptide ABC transporter permease
	Vp_B2_28_0965	0.411	Transcriptional regulator
	Vp_B2_28_0551	0.215	Peptide ABC transporter ATP-binding protein
	Vp_B2_28_0550	0.166	Peptide ABC transporter ATP-binding protein
	Vp_B2_28_3389	0.474	Nucleoid occlusion factor SlmA
	Vp_B2_28_2766	3.787	Bifunctional aspartate kinase/homoserine dehydrogenase II
	Vp_B2_28_4289	0.257	Transcription regulator
	Vp_B2_28_3640	0.241	Electron transporter RnfB
	Vp_B2_28_4302	0.141	Collagenase
	Vp_B2_28_5462	0.331	OtnA protein
	Vp_B2_28_4292	0.310	Amino acid dehydrogenase
Arginine and proline metabolism	*Vp_B2_28_3777*	0.234	Histidine kinase
	Vp_B2_28_2907	70.055	Endonuclease I
	Vp_B2_28_4180	0.283	PTS glucose transporter subunit IIBC
	Vp_B2_28_4181	0.284	Nuclease
	Vp_B2_28_2021	0.077	Lactoylglutathione lyase
	Vp_B2_28_2026	0.091	Glutamine synthetase
	Vp_B2_28_2027	0.137	Hypothetical protein
	Vp_B2_28_2032	0.143	Gamma-glutamylputrescine oxidoreductase

	Vp_B2_28_2030	0.123	Aldehyde dehydrogenase
	Vp_B2_28_2025	0.144	Na+/H+ antiporter family protein
	Vp_B2_28_2028	0.090	Glutamine amidotransferase
	Vp_B2_28_4290	0.198	Aldehyde dehydrogenase
	Vp_B2_28_2031	0.169	Aldehyde dehydrogenase
	Vp_B2_28_0934	0.071	Carboxynorspermidine decarboxylase
Taurine and hypotaurine metabolism	*Vp_B2_28_2494*	0.353	RNA chaperone ProQ
	Vp_B2_28_0127	2.320	Phosphate acetyltransferase
	Vp_B2_28_0126	2.413	Acetate kinase
	Vp_B2_28_2331	3.087	Hypothetical protein
	Vp_B2_28_2493	0.488	Hypothetical protein
	Vp_B2_28_2495	0.420	Tail-specific protease
	Vp_B2_28_4228	0.471	Hypothetical protein
Purine metabolism	*Vp_B2_28_4988*	0.327	Membrane protein
	Vp_B2_28_1503	0.048	ATP-dependent RNA helicase
	Vp_B2_28_1504	0.052	Crp/Fnr family transcriptional regulator
	Vp_B2_28_3533	3.272	Hypothetical protein
	Vp_B2_28_1508	0.054	Sulfate adenylyltransferase subunit 2
	Vp_B2_28_3814	0.351	S-adenosyl-L-homocysteine hydrolase NAD-binding protein
	Vp_B2_28_0927	0.486	Ribonucleotide-diphosphate reductase subunit alpha
	Vp_B2_28_1725	0.350	Molecular chaperone DnaJ
	Vp_B2_28_2334	0.449	RNA helicase
	Vp_B2_28_0222	0.446	Amidophosphoribosyltransferase
	Vp_B2_28_2527	0.375	Hypothetical protein
	Vp_B2_28_2526	0.392	Prepilin-type cleavage/methylation protein
	Vp_B2_28_0493	2.476	Purine-nucleoside phosphorylase
	Vp_B2_28_3625	0.456	Conserved hypothetical protein
	Vp_B2_28_0929	0.367	Ribonucleotide-diphosphate reductase subunit beta
	Vp_B2_28_2411	0.478	Membrane protein
	Vp_B2_28_1500	2.027	Peptidoglycan-binding protein LysM
	Vp_B2_28_1354	3.089	Permease
	Vp_B2_28_5596	0.395	Hypothetical protein
	Vp_B2_28_3305	2.235	tRNA pseudouridine synthase TruD
	Vp_B2_28_5595	0.450	Flagellar sheath protein A
	Vp_B2_28_2529	0.310	Conserved hypothetical protein
	Vp_B2_28_4080	0.496	Patatin family protein
	Vp_B2_28_2528	0.430	Aminobenzoyl-glutamate transporter
	Vp_B2_28_1552	0.495	50S ribosomal protein L27
	Vp_B2_28_4987	0.399	Hypothetical protein
	Vp_B2_28_4549	0.465	Chaperone protein ClpB

alpha-Linolenic acid metabolism	*Vp_B2_28_5043*	0.286	Hypothetical protein
	Vp_B2_28_5133	0.379	Haloacid dehalogenase
	Vp_B2_28_1729	0.494	DNA helicase
Non-alcoholic fatty liver disease	*Vp_B2_28_1595*	0.279	Penicillin-binding protein activator
	Vp_B2_28_1596	0.254	Conserved hypothetical protein
	Vp_B2_28_1597	0.238	DnaA initiator-associating protein DiaA
	Vp_B2_28_5427	2.825	Hypothetical protein
Butanoate metabolism	*Vp_B2_28_3272*	0.464	Type II citrate synthase
	Vp_B2_28_3271	0.420	Phosphoglucomutase, alpha-D-glucose phosphate-specific
	Vp_B2_28_5121	3.969	PrkA family serine protein kinase
	Vp_B2_28_5042	0.398	Redox-sensitive transcriptional activator SoxR
	Vp_B2_28_0249	0.386	Multifunctional fatty acid oxidation complex subunit alpha
	Vp_B2_28_3270	0.391	SeqA protein
	Vp_B2_28_1970	0.189	Hypothetical protein
	Vp_B2_28_1967	0.148	Hypothetical protein
	Vp_B2_28_2291	0.271	Hypothetical protein
	Vp_B2_28_2290	0.413	Hypothetical protein
	Vp_B2_28_3628	0.299	Transcriptional regulator
	Vp_B2_28_3627	0.344	Oxidoreductase

Table S4. Comparison of the major altered metabolic pathways in the three target strains treated by Fraction 2 of *A. villosum* Lour.

**Metabolic Pathway**	**Gram-positive strain**		**Gram-negative strain**
	***S. aureus* GIM1.441**	***B. cereus* Y1**	***V. parahemolyticus* B2-28**
Valine, leucine and isoleucine biosynthesis	+	-	-
Biosynthesis of various other secondary metabolites	+	-	-
Ascorbate and aldarate metabolism	+	-	-
Ribosome	+	-	-
C5-Branched dibasic acid metabolism	+	-	-
Alanine, aspartate and glutamate metabolism	+	-	-
O-Antigen nucleotide sugar biosynthesis	+	+	-
Biosynthesis of various antibiotics	+	-	-
Bacterial chemotaxis	-	+	-
Nucleotide excision repair	-	+	-
Histidine metabolism	-	+	-
Mismatch repair	-	+	-
Valine, leucine and isoleucine degradation	-	+	-
Biosynthesis of siderophore group nonribosomal peptides	-	+	-
Glyoxylate and dicarboxylate metabolism	-	-	+
Propanoate metabolism	-	-	+

Lysine degradation	-	-	+
Fatty acid degradation	-	-	+
Carbon fixation pathways in prokaryotes	-	-	+
One carbon pool by folate	-	-	+
Methane metabolism	-	-	+
Sulfur metabolism	-	-	+
ABC transporters	-	-	+
Quorum sensing	-	-	+
Arginine and proline metabolism	-	-	+
Taurine and hypotaurine metabolism	-	-	+
Purine metabolism	-	-	+
alpha-Linolenic acid metabolism	-	-	+
Non-alcoholic fatty liver disease	-	-	+
Butanoate metabolism	-	-	+
Central carbon metabolism in cancer	-	-	+

Note: +: altered. -: not altered.

Table S5. The oligonucleotide primers designed and used in the RT-qPCR assay.

Bacterial Strain	Primer	Sequence (5′ to 3′)
S. aureus GIM1.441	*B4602_RS10775-F*	AAATACCGCAAGAACCCAA
	B4602_RS10775-R	GCGATACGCTGAACACTCG
	B4602_RS01530-F	TACCGCAAGAACCCAAGA
	B4602_RS01530-R	CGATACGCTGAACACTCG
	B4602_RS02195-F	GTGTTGAACGAGCGACCAG
	B4602_RS02195-R	AACGCATCTTCCAAATCCC
	B4602_RS00385-F	CAATTACGATAGAGGGAAGG
	B4602_RS00385-R	TACACTTGATAATCCGCTTC
	B4602_RS00410-F	TTTTGGTGGCCCTGGTAAG
	B4602_RS00410-R	TTGGGAAATGGTCCTGCTA
B. cereus Y1	*EJ379_25705*-F	GCAAGAACCCAAGAAACTAT
	EJ379_25705-R	CAATGACTTTATTCCCATCA
	EJ379_05155-F	CTGAGATGTTCCGTAACCCTG
	EJ379_05155-R	GTTGCGTAAATAACCGACTT
	EJ379_01760-F	GCAGCAGCAGGTTCAATA
	EJ379_01760-R	GAAGGGCAACATCATCTA
	EJ379_19260-F	CGCCCTGCTTCAGTCGTA
	EJ379_19260-R	ACTCGGCAATGCCTCACC
	EJ379_27830-F	CGGAAATGACTATCCAACGA
	EJ379_27830-R	ACCTGGGCGACGCTCTGTAA
V. parahaemolyticus B2-28	*Vp_B2_28_4316*-F	AGGTGGTTTCCCAGGTGC
	Vp_B2_28_4316-R	CCCATTGATACTGGCTTACC
	Vp_B2_28_2625-F	CAAGTGGAAGTGCGTGAA
	Vp_B2_28_2625-R	GTGCCTGGAAGACCGATA
	Vp_B2_28_2812-F	CACCTTACCGTTGGATTG
	Vp_B2_28_2812-R	CTGGCAGTTTGAAGTTATTG
	Vp_B2_28_0737-F	TGTTGCTCAAGCGAAAGAA

Vp_B2_28_0737-R	GAATAATGCCATCCCAATC
Vp_B2_28_0737-F	GTTATCTCCATCATTGGCTCT
Vp_B2_28_0737-R	CGAACGAATACGCTGAAC

Table S6. The relative expression of the representative DEGs by the RT-qPCR assay.

Bacterial Strain	Gene	Predicted Protein	Fold Change	
			RNA-Seq.	RT-qPCR
S. aureus GIM1.441	*B4602_RS10775*	Ketol-acid reductoisomerase	0.447	0.236
	B4602_RS01530	PTS ascorbate transporter subunit IIC	8.846	1.532
	B4602_RS02195	Glutamate synthase large subunit	2.141	1.901
	B4602_RS00385	Staphyloferrin B biosynthesis protein SbnC	2.88	5.375
	B4602_RS00410	Staphyloferrin B biosynthesis decarboxylase SbnH	2.571	3.309
B. cereus Y1	*EJ379_25705*	Methyl-accepting chemotaxis protein	0.136	0.097
	EJ379_05155	Protein-glutamate O-methyltransferase CheR	0.312	0.377
	EJ379_01760	NAD-dependent DNA ligase LigA	0.26	0.075
	EJ379_19260	DNA mismatch repair endonuclease MutL	0.332	0.694
	EJ379_27830	UDP-glucose 4-epimerase GalE	2.904	3.320
V. parahaemolyticus B2-28	*Vp_B2_28_4316*	Multidrug transporter AcrB	0.126	0.041
	Vp_B2_28_2625	RpoS	2.64	7.21
	Vp_B2_28_2812	Phage shock protein G	0.098	0.549
	Vp_B2_28_0737	Amino acid transporter	0.201	0.033
	Vp_B2_28_0735	Serine protease	0.221	0.652

Table S7. The bacterial strains and media used in this study.

Bacterial Strain	Culture Medium	Source
Aeromonas hydrophila ATCC35654	TSB	ATCC, United States
Bacillus cereus Y1	TSB	LS-SHOU, China
Enterobacter cloacae ATCC13047	TSB	Biobw, China
Enterobacter cloacae D1	TSB	Biobw, China
Escherichia coli ATCC8739	TSB	Biobw, China
Escherichia coli K12	TSB	IIM, China
Escherichia coli ATCC25922	TSB	IIM, China
Enterobacter sakazakii CMCC45401	TSB	Biobw, China
Klebsiella pneumoniae 7-17-16	EE	LS-SHOU, China
Pseudomonas aeruginosa ATCC9027	TSB	Biobw, China
Pseudomonas aeruginosa ATCC27853	TSB	Biobw, China
Staphylococcus aureus GIM1.481	TSB	ATCC, United States
Staphylococcus aureus GIM1.441	TSB	ATCC, United States
Staphylococcus aureus GIM1.160	TSB	ATCC, United States
Shigella dysenteriae CMCC51252	TSB	GCCC, China
Salmonella choleraesuis ATCC13312	TSB	ATCC, United States
Shigella flexneri GIM1.238	TSB	GCCC, China
Shigella flexneri GIM1.539	TSB	GCCC, China

Shigella flexneri GIM1.231	TSB	GCCC, China
Salmonella paratyphi GIM1.235	TSB	GCCC, China
Shigella sonnei GIM1.424	TSB	ATCC, United States
Shigella sonnei GIM1.239	TSB	ATCC, United States
Salmonella enterica subsp. *enterica* (*ex* Kauffmann and Edwards) Le Minor and Popoff serovar Vellore ATCC15611	LB	LS-SHOU, China
Vibrio cholerae GIM1.449	TSB	GCCC, China
Vibrio cholerae B1	TSB	LS-SHOU, China
Vibrio parahaemolyticus B2-28	TSB	LS-SHOU, China
Vibrio parahaemolyticus N2-5	TSB	LS-SHOU, China
Vibrio parahaemolyticus N9-20	TSB	LS-SHOU, China
Vibrio alginolyticus ATCC33787	TSB	ATCC, United States
Vibrio fluvialis ATCC33809	Marine 2216	ATCC, United States
Vibrio harveyi ATCC BAA-1117	Marine 2216	ATCC, United States
Vibrio harveyi ATCC33842	Marine 2216	ATCC, United States
Vibrio metschnikovi ATCC700040	TSB	LS-SHOU, China
Vibrio mimicus bio-56759	Marine 2216	ATCC, United States
Vibrio parahaemolyticus ATCC17802	TSB	ATCC, United States
Vibrio parahaemolyticus ATCC33847	TSB	ATCC, United States
Vibrio vulnificus ATCC27562	Marine 2216	Biobw, China

Note: ATCC: American Type Culture Collection, United States; GCCC, Guangdong Culture Collection Center, Guangzhou, China; IIM, Institute of Industrial Microbiology, Shanghai, China; LS-SHOU, Laboratory stock, Shanghai Ocean University, Shanghai, China.

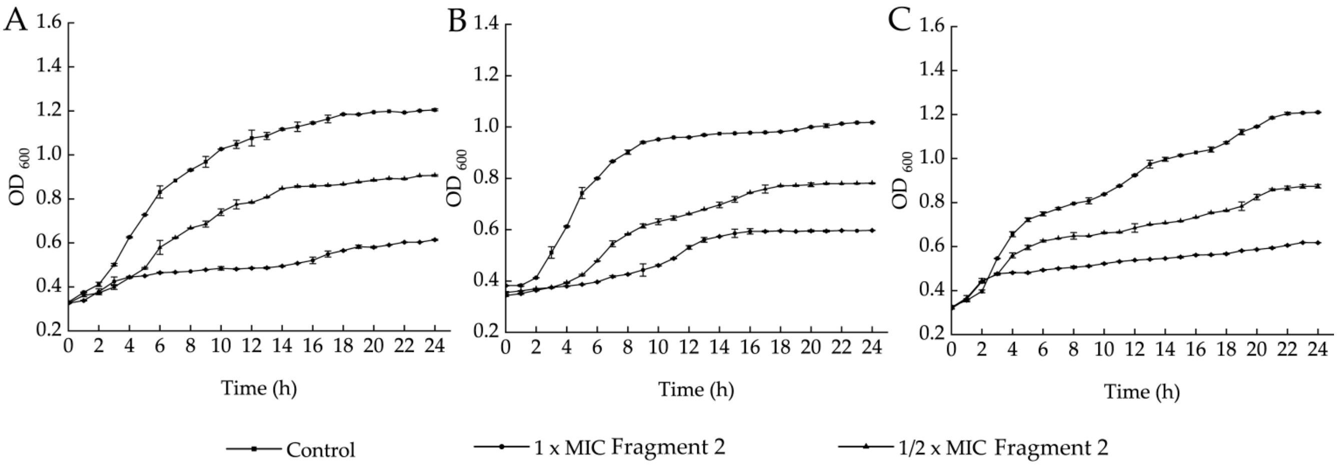

Figure S1. Growth curves of target strains treated by Fraction 2 from *A. villosum* Lour. (A—C): *S. aureus* GIM1.441, *B. cereus* Y1, and *V. parahemolyticus* B2-28, respectively. The strains were incubated in the TSB medium at 37 °C.

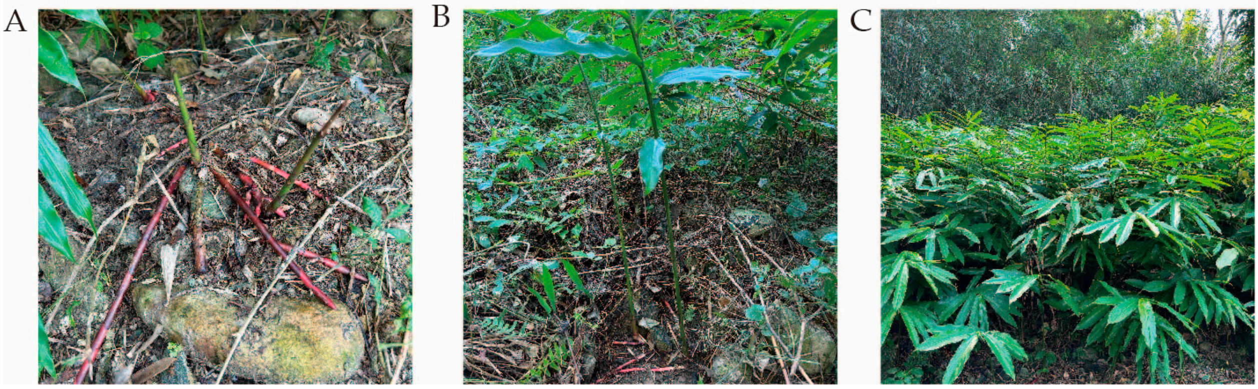

Figure S2. The plant of *A. villosum* Lour. (A–C): the different plant growth period.
